# The gut virome is associated with stress-induced changes in behaviour and immune responses in mice

**DOI:** 10.1038/s41564-023-01564-y

**Published:** 2024-02-05

**Authors:** Nathaniel L. Ritz, Lorraine A. Draper, Thomaz F. S. Bastiaanssen, Christopher J. R. Turkington, Veronica L. Peterson, Marcel van de Wouw, Klara Vlckova, Christine Fülling, Katherine E. Guzzetta, Aurelijus Burokas, Hugh Harris, Marion Dalmasso, Fiona Crispie, Paul D. Cotter, Andrey N. Shkoporov, Gerard M. Moloney, Timothy G. Dinan, Colin Hill, John F. Cryan

**Affiliations:** 1 APC Microbiome Ireland, University College Cork, Cork, Ireland; 2 Department of Anatomy and Neuroscience, University College Cork, Cork, Ireland; 3 School of Microbiology, University College Cork, Cork, Ireland; 4 Department of Food Biosciences, Teagasc Food Research Centre, Moorepark, Fermoy, Ireland; 5 Department of Psychiatry and Neurobehavioural Science, University College Corke, Cork, Ireland; 6 Present Address: Department of Pediatrics, University of Calgary, Calgary, Canada; 7 Present Address: Department of Biosystems Science and Engineering, ETH Zürich, Basel, Switzerland; 8 Present Address: Department of Biological Models, Institute of Biochemistry, Life Sciences Center, Vilnius University, Vilnius, Lithuania; 9 Present Address: Normandie Univ, UNICAEN, UNIROUEN, ABTE, 14000, Caen, France

**Keywords:** Bacteriophages, Neuroscience, Microbiome

## Abstract

The microbiota–gut–brain axis has been shown to play an important role in the stress response, but previous work has focused primarily on the role of the bacteriome. The gut virome constitutes a major portion of the microbiome, with bacteriophages having the potential to remodel bacteriome structure and activity. Here we use a mouse model of chronic social stress, and employ 16S rRNA and whole metagenomic sequencing on faecal pellets to determine how the virome is modulated by and contributes to the effects of stress. We found that chronic stress led to behavioural, immune and bacteriome alterations in mice that were associated with changes in the bacteriophage class *Caudoviricetes* and unassigned viral taxa. To determine whether these changes were causally related to stress-associated behavioural or physiological outcomes, we conducted a faecal virome transplant from mice before stress and autochthonously transferred it to mice undergoing chronic social stress. The transfer of the faecal virome protected against stress-associated behaviour sequelae and restored stress-induced changes in select circulating immune cell populations, cytokine release, bacteriome alterations and gene expression in the amygdala. These data provide evidence that the virome plays a role in the modulation of the microbiota–gut–brain axis during stress, indicating that these viral populations should be considered when designing future microbiome-directed therapies.

## Abstract (web summary)

The gut virome is altered in a mouse model of chronic stress and is associated with changes in behavioural and immune response, which can be restored using faecal virome transfer.

## Main

Stress-related psychiatric disorders such as depression and anxiety are among the highest in lifetime prevalence across the globe and are significant societal burdens^1–3^. Thus, understanding the biological consequences of chronic stress is an important avenue in developing novel strategies for stress-related disorders. Increasing evidence suggests that brain–gut interactions may gate the response to stress^4^. In tandem, the gut microbiota—the community of microorganisms including bacteria, viruses, archaea, protozoas and fungi that inhabit the intestine—has been shown to influence the development and function of the immune and nervous systems through the microbiota–gut–brain axis^2,5^. Bacteria make up the vast majority and metabolic capability of the cellular microorganisms in the gut and have been shown to modulate stress responses^6^. Although less studied, intestinal viruses, which are predominantly composed of bacteriophages (or phages), infect bacteria and can multiply and lyse or incorporate into their host genome and replicate alongside them^7,8^. These phages can modulate the structure and function of the microbiota and contribute towards diversity, stability and resilience of microbiota at the community level^9^. Faecal virome transplants (FVT) prepared from faecal filtrate, composed largely of viral (primarily phage) particles and devoid of cellular microbes, have been used as microbiota-based treatments to improve clinical symptoms of *Clostridioides difficile* infection and preclinical models of type 2 diabetes, antibiotic-induced microbiota perturbation, necrotizing enterocolitis and small intestinal bacterial overgrowth^10–14^.

Numerous stress-related disorders have been associated with alterations in the gut bacteriome^15–18^, and there is evidence that the transfer of bacterial microbiota from patients with major depression and general anxiety disorders to rodents can transfer symptoms related to the phenotypes^19–21^. As the virome is intimately linked to the composition of the bacteriome^9^, it also changes in response to the environment and psychological state of the host^18,22–24^. Recently, phages from the class *Caudoviricetes* have been linked with executive function of the host across species, which supports the theory that viral members of the microbiota can impact host behaviour^25^. While the gut bacteriome has gained attention and is currently the subject of growing research, the dynamic involvement of how the virome interacts with its bacterial hosts and how both entities, in conjunction, affect stress-related health and disease status is largely unexplored. Here we assess the impact of stress on the virome and demonstrate that an autochthonous (that is, indigenous origin) virome transfer can prevent the manifestation of stress-related behavioural, immune and neurobiological changes in mice.

## Results

**Extended Data Fig. 1:**
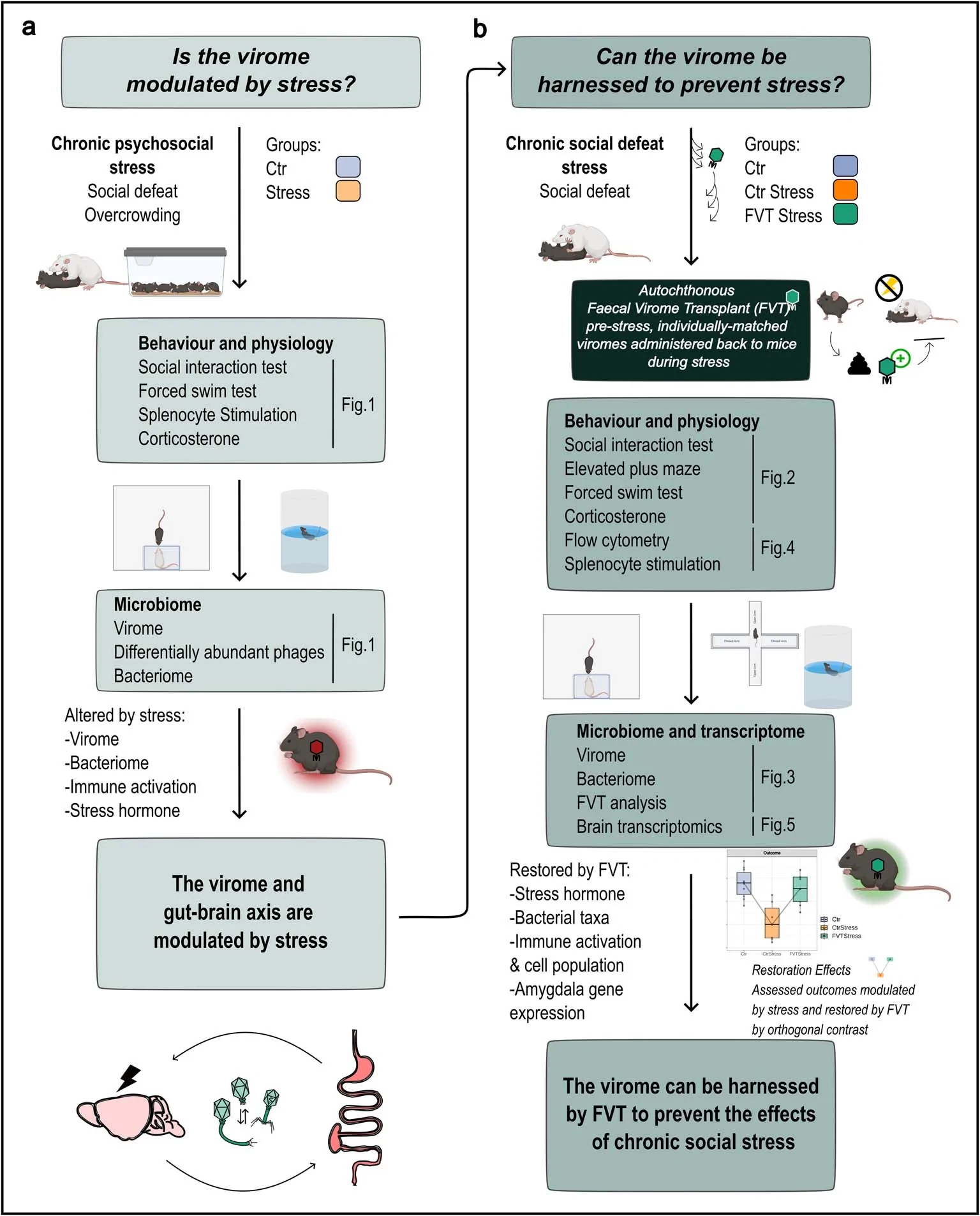
Study workflow summary. (**a**) First, to answer whether the gut virome is affected by stress we assessed behaviour, physiology, and microbiome alterations following chronic psychosocial stress. (**b**) Then, to determine if the virome can be harnessed to prevent stress, we administered an autochthonous faecal virome transplant (FVT) treatment to mice as they underwent stress and found that the FVT attenuated stress effects on behaviour, physiology, microbiome, and brain transcriptomics.

A workflow summary to outline how the virome is modulated by stress and how the virome was leveraged to prevent the effects of stress is provided in Extended Data Fig. 1a,b.

### The virome and gut–brain axis are modulated by stress

**Fig. 1:**
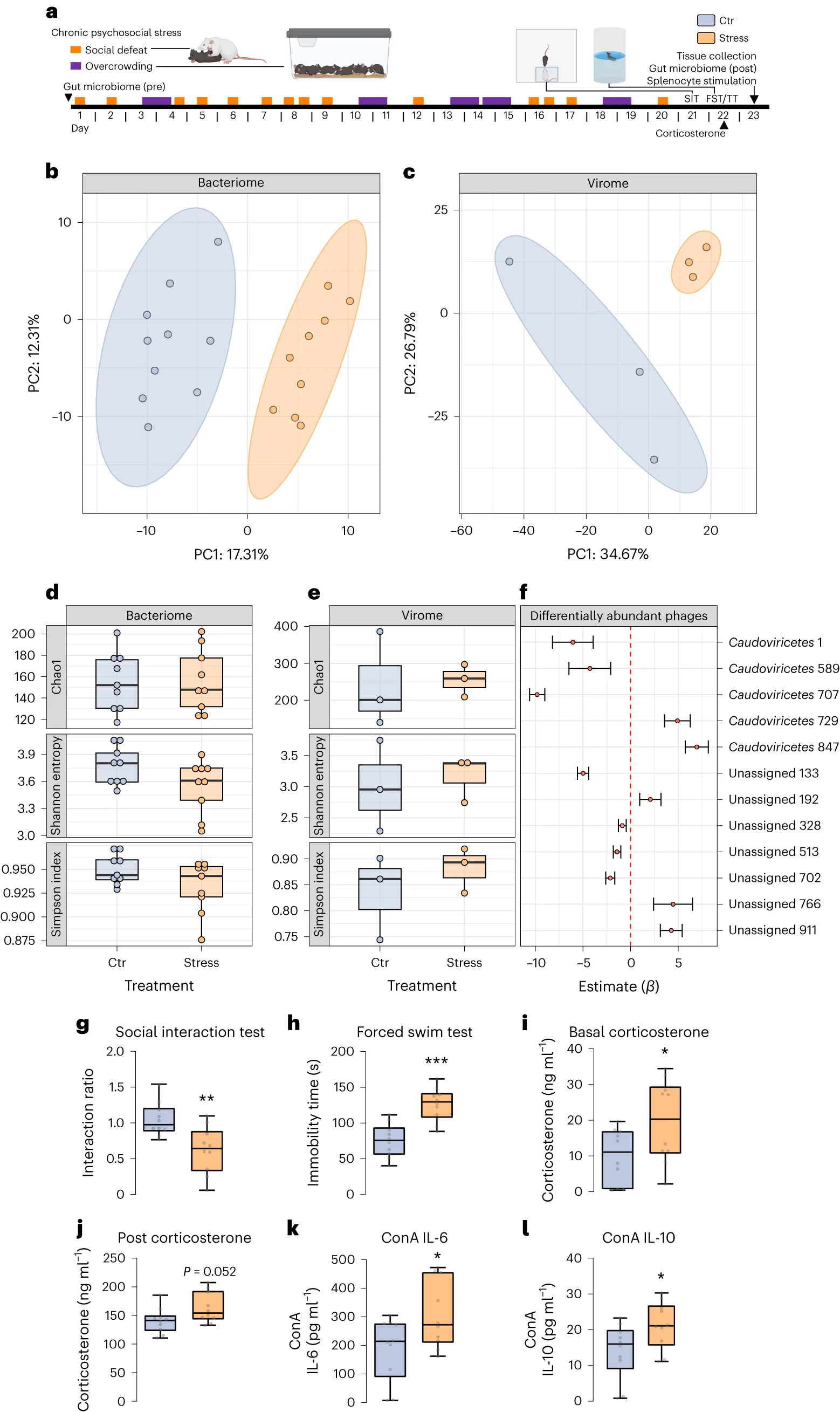
Chronic psychosocial stress leads to microbiome, behavioural, physiological and immune alterations. **a**, Chronic psychosocial stress experimental timeline. **b**, Faecal bacteriome Aitchison beta-diversity was significantly different between stress (Stress) and control (Ctr) groups as assessed by PERMANOVA (*R*^2^ = 0.1693, *P* < 0.01; Ctr *n* = 10, Stress *n* = 9). **c**, There was a trend of a difference in pooled faecal virome Aitchison beta-diversity between stress and control groups as assessed by PERMANOVA (*R*^2^ = 0.3011, *P* = 0.1; Ctr *n* = 10 pooled in 3 samples, Stress *n* = 10 pooled in 3 samples). **d**,**e**, No differences were found in bacteriome (**d**) (Ctr *n* = 9, Stress *n* = 9) or virome (**e**) alpha-diversity metrics (Ctr *n* = 10 pooled in 3 samples, Stress *n* = 10 pooled in 3 samples). **f**, Differentially abundant phages of the stress group compared to the control group identified at the class taxonomic level; data are presented as mean ± 95% confidence intervals of *β*-estimate (Ctr *n* = 10 pooled in 3 samples, Stress *n* = 10 pooled in 3 samples). **g**, In the social interaction test (SIT), stress significantly reduced social interaction as measured by percentage of time in the interaction zone with CD1 mice divided by the time spent in the interaction zone without CD1 mice (*t*(18) = 3.317, *P* < 0.01; Ctr *n* = 10, Stress *n* = 10). **h**, In the FST test, stress-coping behaviour measured by time spent immobile was significantly increased by stress (*t*(18) = −4.946, *P* < 0.001; Ctr *n* = 10, Stress *n* = 10). **i**, Stress caused a significant increase in basal plasma corticosterone collected by tail tipping (TT) before FST (*t*(15.68) = −2.224, *P* < 0.05; Ctr *n* = 10, Stress *n* = 10). **j**, There was a trend for corticosterone measured at 45 min post FST to be increased in the stress group (*t*(18) = −2.077, *P* = 0.052; Ctr *n* = 10, Stress *n* = 10). **k**,**l**, Analysis of inflammatory cytokines collected from supernatant after 24 h ex vitro ConA splenocyte stimulations of IL-6 (**k**) and IL-10 (**l**) revealed significant increases in the stress group compared with the control (*t*(18) = −2.316, *P* < 0.05; *t*(18) = −2.257, *P* < 0.05, respectively; Ctr *n* = 10, Stress *n* = 10). Data in **d**–**l** were compared using independent-samples two-sided *t*-test (*P < 0.05, **P < 0.01, ***P < 0.001). Data presented as boxplots display the minimum, first quartile, median, third quartile and maximum.

**Extended Data Fig. 2:**
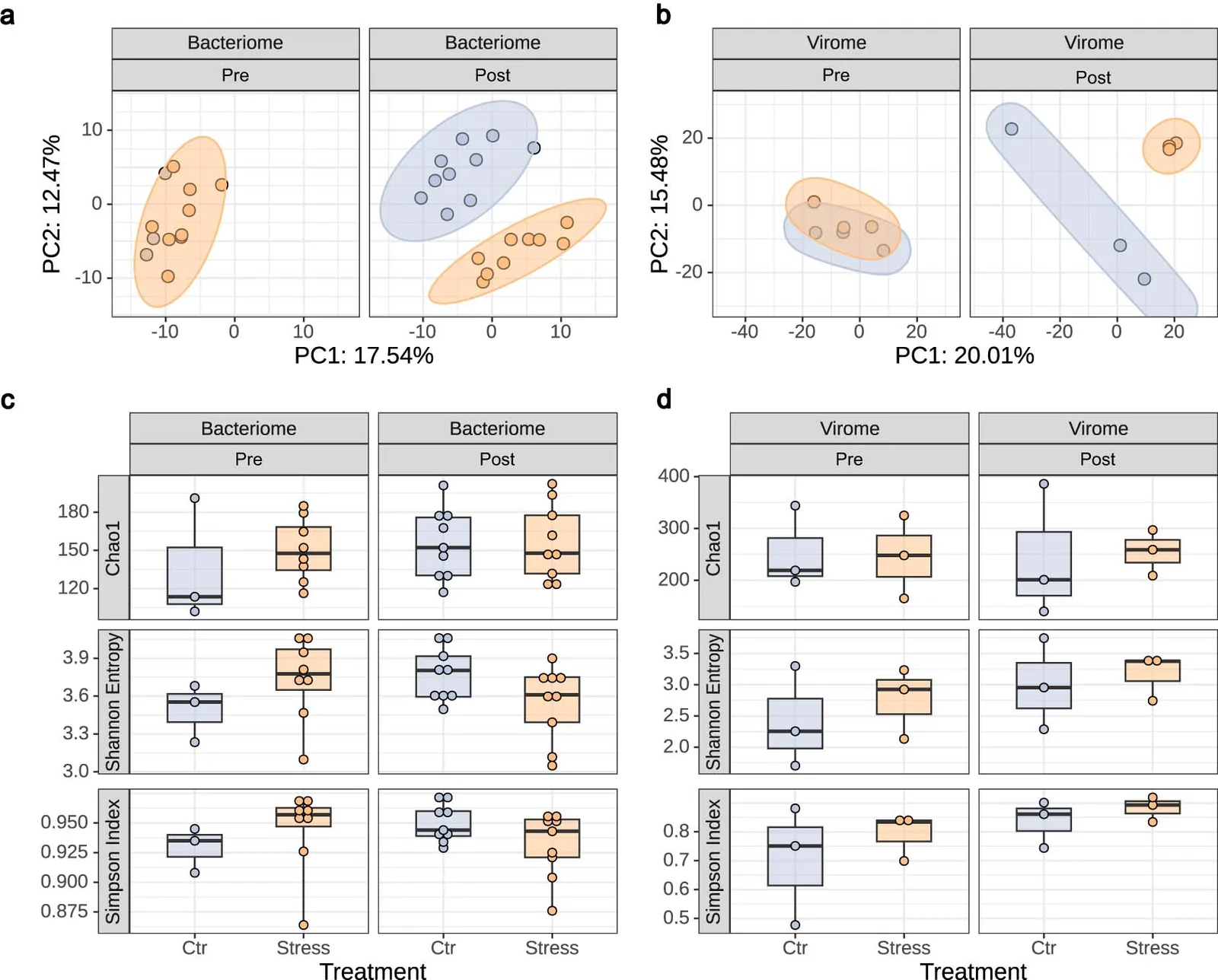
Before and after chronic psychosocial stress microbiome comparisons. (**a**) Bacteriome Aitchison beta diversity measures before (Pre) and after (Post) chronic psychosocial stress, no differences were found in Pre, however there was a significant alteration found in Post assessed by PERMANOVA (R^2^ = 0.1693, *p* < 0.01) (*n* = 10 Ctr, pooled in 3 in Pre timepoint; *n* = 9 Stress). (**b**) Virome Aitchison beta diversity measures before (pre) and after (post) chronic psychosocial stress, no differences were found in Pre, however there was a trend for alteration found in Post assessed by PERMANOVA (R^2^ = 0.3011, *p* = 0.1) (*n* = 10 Ctr pooled in 3; *n* = 10 Stress pooled in 3). (**c**) Bacteriome alpha diversity measures (Chao1, Shannon Entropy, and Simpson Index), no significant differences were found Pre or Post assessed by independent-samples two-sided T test (*n* = 10 Ctr, pooled in 3 in Pre timepoint; *n* = 9 Stress). (**d**) Virome alpha diversity measures (Chao1, Shannon Entropy, and Simpson Index), no significant differences were found Pre or Post assessed by independent-samples two-sided T test (*n* = 10 Ctr pooled in 3; *n* = 10 Stress pooled in 3). Data presented as box plots display the minimum, first quartile, median, third quartile, and maximum.

First, mice underwent chronic social stress (chronic psychosocial defeat stress; that is, unpredictable chronic social defeat and single housing with periods of overcrowding), while control mice were similarly handled and group housed, then stress-coping behaviour, immune modulation and microbiome composition were tested (Fig. 1a). To assess the impact of chronic social stress on virome and bacteriome composition, viral metagenomic sequencing was combined with 16S bacterial ribosomal (r)RNA gene sequencing on faecal pellets before (pre) and following the imposition of stress (post; Extended Data Fig. 2a–d). Faecal bacteriome Aitchison beta-diversity was significantly altered by stress (Fig. 1b). Qualitatively, stress markedly affected virome composition at the beta-diversity level but due to the use of pooled groups, this only showed a trend between groups despite a clear visual separation (Fig. 1c). For the virome analysis, groups of *n* = 10 and 9 (Ctr and Stress, respectively) were pooled to ensure adequate nucleic acid content. Bacterial and viral alpha-diversity metrics were not affected by stress (Fig. 1d, e). However, 12 phages were found to be differentially abundant following stress, 5 of which were identified as class *Caudoviricetes* (Fig. 1f).

Chronic social stress affected behaviour and immune responses in line with previous murine research^26–28^. There was a distinct reduction in social interaction behaviour following stress (Fig. 1g). Immobility times in the forced swim test (FST) and, correspondingly, basal corticosterone concentrations were increased in the stress recipient group in addition to a trend of increased corticosterone post FST (Fig. 1h–j). In addition, there was increased inflammatory cytokine release (IL-6 and IL-10) from concanavalin A (ConA)-stimulated splenocytes (Fig. 1k,l), as commonly seen following chronic social stress in mice (Extended Data Table 1a)^29,30^. Increased inflammatory cytokine release from splenocyte cultures following stress has been previously linked with anxiety-like behaviour^29^ and stimulation with ConA indicates humoral-based immune activation capable of driving inflammation.

### Stress behaviour is attenuated by faecal virome transplant

**Fig. 2:**
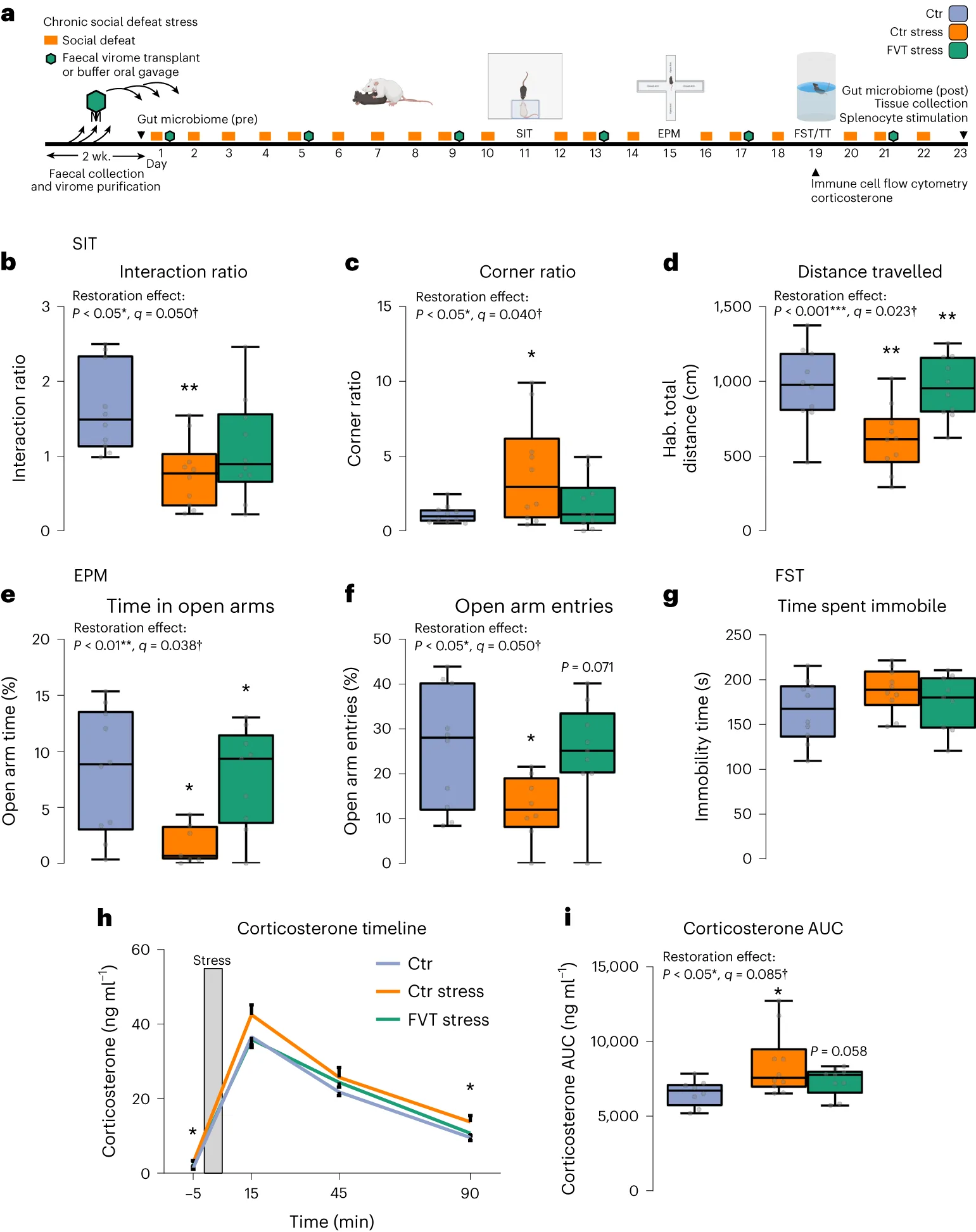
Faecal virome transplant attenuates stress-related behaviours. **a**, Experimental timeline. **b**, In the SIT test, stress significantly reduced the social interaction ratio in the Ctr Stress group (*P* < 0.05, *q* = 0.050, *t*(27) = 9.395, *P* < 0.01, *P* = 0.427, respectively; *n* = 10 per group). **c**, The corner ratio was significantly increased by stress (*P* < 0.05, *q* = 0.040, *t*(27) = 6.067, *P* < 0.05, *P* = 0.111; *n* = 10 per group). **d**, The distance travelled during the habituation phase of the test was significantly reduced by stress and restored in the FVT group (*P* < 0.001, *q* = 0.023, *t*(27) = 17.761, *P* < 0.01, *P* < 0.01; *n* = 10 per group). **e**, In the EPM test, stress significantly increased the percentage of time spent in the open arms and the FVT restored this effect (*P* < 0.01, *q* = 0.038, *t*(23) = 4.818, *P* < 0.05, *P* < 0.05; Ctr *n* = 10, Ctr Stress *n* = 7, FVT Stress *n* = 9). **f**, Entries into the EPM open arms were reduced in the stress group followed by a trend of restoration by FVT (*P* < 0.05, *q* = 0.050, *t*(23) = 7.254, *P* < 0.05, *P* = 0.071; Ctr *n* = 10, Ctr Stress *n* = 7, FVT Stress *n* = 9). **g**, In the FST, there were no effects recorded. **h**, Plasma corticosterone collected by TT was measured at −5 min (basal), +15 min, +45 min and +90 min from FST initiation; data are presented as mean ± s.e.m. There was increased corticosterone in the Ctr Stress group compared with the Ctr at the basal and 90 min post-FST timepoints as assessed by orthogonal contrast followed by Tukey’s two-sided pairwise post hoc test (*P* < 0.05, *t*(16) = 2.673, *P* < 0.05, *t*(16) = 2.386, *P* < 0.05, respectively; Ctr *n* = 8, Ctr Stress *n* = 10, FVT Stress *n* = 9). **i**, Area-under-curve (AUC) of corticosterone was increased in the stress group followed by a trend of reduction by FVT (*P* < 0.05, *q* = 0.085, *t*(26) = 26.703, *P* < 0.05, *P* = 0.058; Ctr *n* = 8, Ctr Stress *n* = 10, FVT Stress *n* = 9). In **b**–**g** and **i**, the restoration effect was measured using planned orthogonal contrast followed by Tukey’s post hoc test (two-sided pairwise comparisons of Ctr–Ctr Stress and Ctr Stress–FVT Stress) and BH-FDR adjustment. Data are presented in the following order: *P* = restoration effect measured by orthogonal contrast; *q* = BH-FDR-adjusted *P* value; *P* = Tukey’s post hoc test. Tukey’s pairwise post hoc *P* values were calculated for Ctr–Ctr Stress and Ctr Stress–FVT Stress. Significant effects are denoted by: *P < 0.05, **P < 0.01, ***P < 0.001, †*q* < 0.2. Data presented as boxplots display the minimum, first quartile, median, third quartile and maximum.

Following the observation that chronic social stress alters behaviour and the population structure of both bacteriome and virome, we next sought to determine whether a virome intervention could modulate the microbiota and improve stress-related behavioural sequelae. To accomplish this, we collected faeces from healthy non-stressed mice, purified the viral fraction and prepared it for subsequent administration to animals via oral gavage. Then, we subjected the same animals to chronic social defeat stress (while housed singly) with and without autochthonous faecal virome transplant (FVT). We removed the overcrowding periods of the stress model to prevent coprophagia, as the specificity of the FVT would be hindered if there were additional microbiota crossover events. After 10 d of stress and treatment, animals were subjected to behaviour tests to assess social (via a social interaction test (SIT)), anxiety-like (via elevated plus maze (EPM) test) and stress-coping (via FST test) behaviours while conjointly going through the stress paradigm (Fig. 2a). We confirmed the behavioural stress phenotype in control mice (receiving a buffer oral gavage) and additionally found that there was a restoration effect of stress-induced social investigatory deficits by stress and FVT treatment (Fig. 2b,c), in addition to significant improvement in locomotor (Fig. 2d) and anxiety-like behaviours (Fig. 2e,f). No stress effects were observed in immobility time during the forced swim test (a measure of stress-coping behaviour; Fig. 2g); we attribute this to the co-administration of the buffer solution to the unstressed controls by oral gavage in an attempt to account for handling stress effects. However, we did observe increased circulating stress hormone corticosterone in the stress group at basal level and 90 min post stress, and a restoration effect in the area under the curve data in response to the forced swim test stressor (Fig. 2h,i).

### FVT inocula and virome characterization

FVT is a technique where free viral particles, which are dominated by phages, are extracted from the rest of the microbiota and other particulate material. By using phages to target the bacteriome, this treatment both specifically targets the microbiota–gut–brain axis and is safer than comparable faecal microbiota transplants due to the removal of live cellular microorganisms. FVT has previously been used to specifically target the murine bacteriome with phages from another source^11–14^. Phage infection of bacterial hosts consists of highly specific targeting in most cases, being governed by strain-level precision. This contributes to the high inter-individual variability of the phageome^22,31–33^. We used an autochthonous FVT model since it theoretically provides the highest probability of active phage infections of bacteriome members due to high accordance between phage–host pairs.

**Fig. 3:**
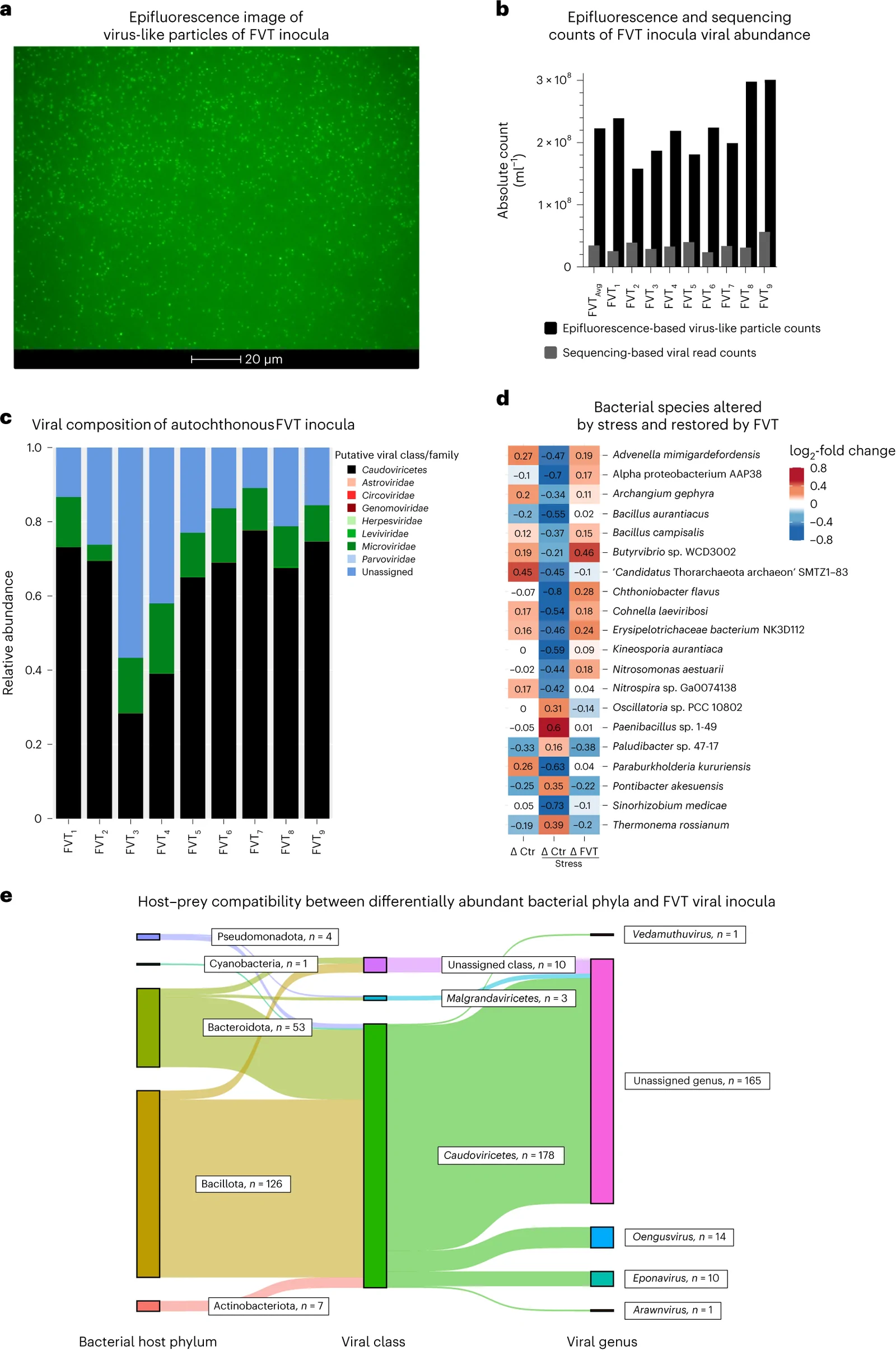
Viral characterization of the faecal virome transplant and the effect on recipient microbiome. **a**, Representative epifluorescence microscopy image of virus-like particles from an FVT inoculum stained with SYBR Gold at ×100 magnification. No other microbes were observed using this method and viral counts were assessed for each FVT inoculum (FVT Stress *n* = 9). For quantification, 4 representative images per sample were taken at predefined quadrant fields of view of the slide and the mean count was used to calculate total number of virus-like particles per sample. **b**, Absolute counts of virus-like particles calculated using epifluorescence microscopy (black) and sequencing reads (grey) using a Q33 *Lactococcal* phage spike (FVT Stress *n* = 9). **c**, Autochthonous FVT inocula relative abundance of viral taxa for each individual FVT treatment (FVT Stress *n* = 9). **d**, Longitudinal measure of differential bacterial species affected by stress and restored by FVT study as assessed by linear mixed effect models (Ctr *n* = 10, Ctr Stress *n* = 8, FVT Stress *n* = 9). **e**, Predicted phage–host pairs present in FVT inocula and faecal bacteriome phyla of the FVT Stress group analysed at the end of study (FVT Stress *n* = 9).

Images of FVT samples were taken and confirm that the FVT inocula were free of larger microorganisms (representative image depicted in Fig. 3a). Approximately 2.2 × 10^8^ virus-like particles per ml were counted in the FVT solution (Fig. 3b). Furthermore, the FVT solution was characterized by virome sequencing (with known concentrations of lactococcal phage Q33 spiked to calculate absolute abundance) and ∼3.5 × 10^7^ plaque-forming units per ml viruses were enumerated. The incongruity in count data metrics can be explained by the methods of detection as sequencing-based read enumeration is limited by the requirement of next generation sequencing library preparation methods to match viral contigs to a known viral gene, whereas, the epifluorescence microscopy method functions through SYBR staining of FVT inocula samples where nucleic acids fluoresce upon binding. However, the presence of larger cellular microorganisms was not detected by either of the FVT inocula characterization approaches.

**Extended Data Fig. 3:**
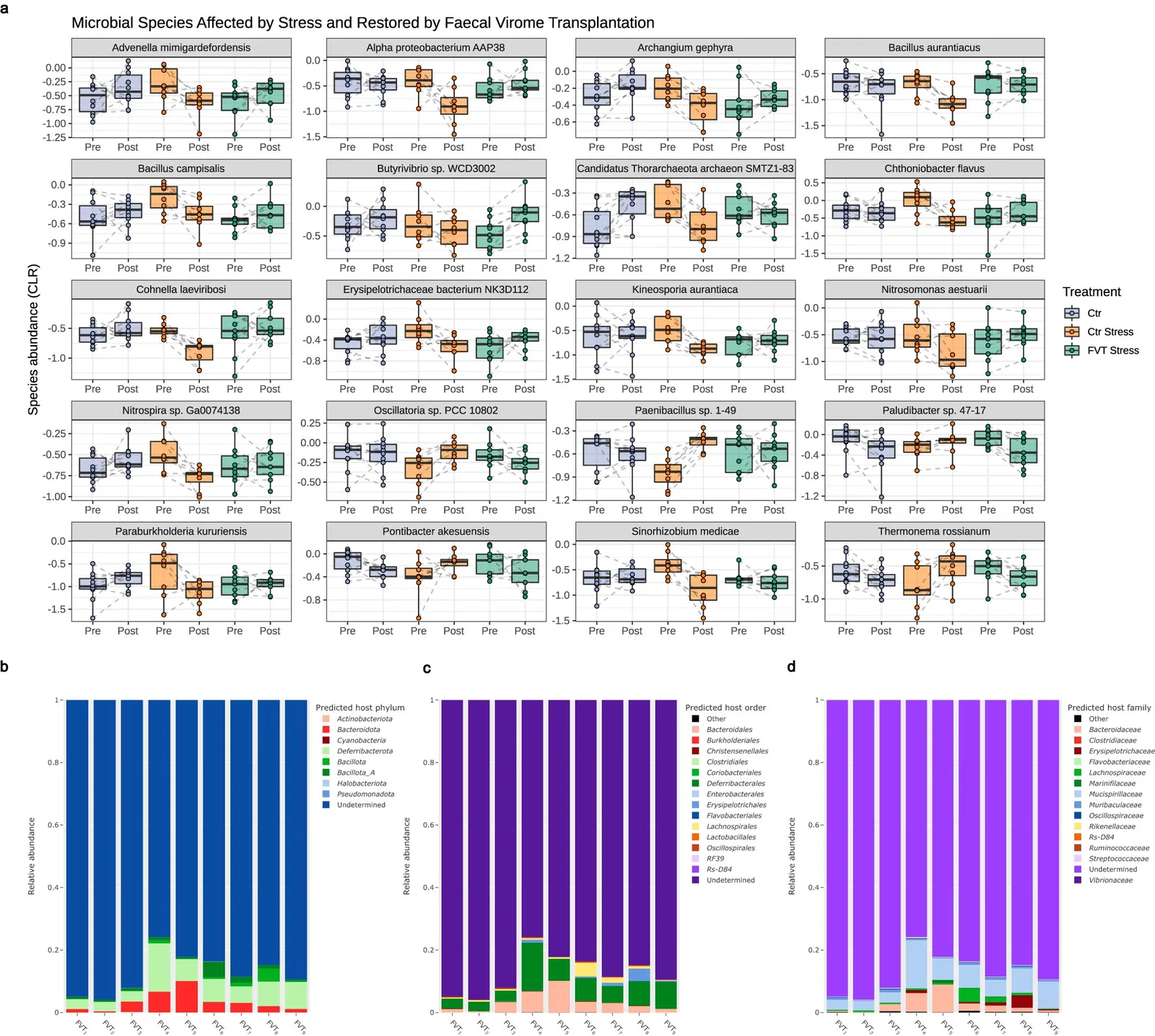
Bacterial species altered by stress and restored by FVT and predicted host taxa of FVT inocula. (**a**) Differentially abundant bacterial species affected by stress and restored by FVT before (Pre) and after (Post) study compared by a linear mixed effects model followed by. Storey’s FDR testing (Ctr *n* = 10, Ctr Stress *n* = 8, FVT Stress *n* = 9). (**b–d**) Predicted phage hosts of FVT inocula at phyla (b), order (c), and family levels (d) (FVT Stress *n* = 9). Data presented as box plots display the minimum, first quartile, median, third quartile, and maximum.

**Extended Data Fig. 4:**
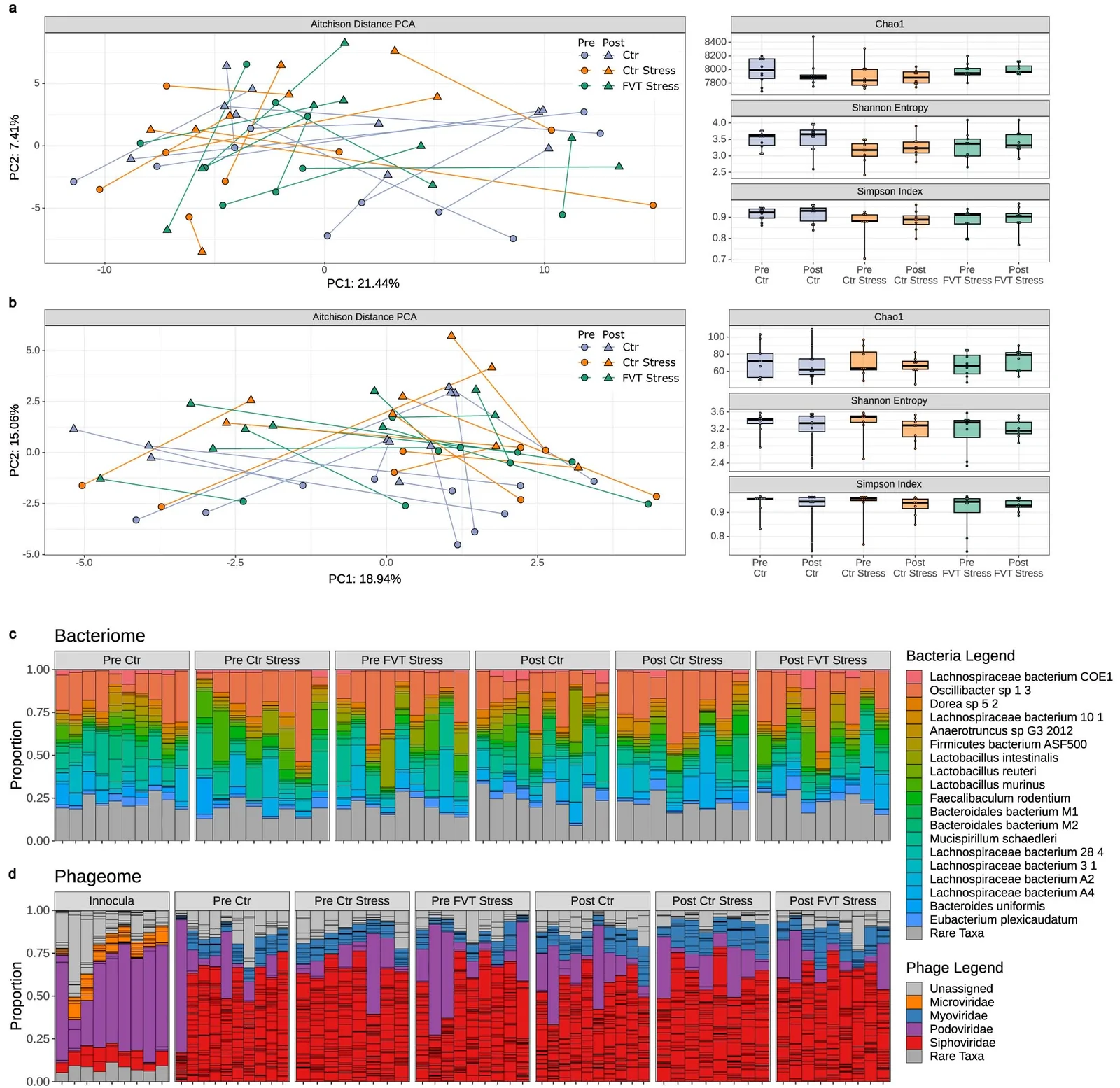
Diversity and community microbiome measures following chronic stress and FVT. (**a**, **b**) Aitchison beta diversity distance, Shannon entropy, and Simpson index of bacteriome (a) and virome (b) (Ctr *n* = 10, Ctr Stress *n* = 8, FVT Stress *n* = 9). (**c, d**) Faecal bacteriome (c) and FVT inocula and faecal virome (d) community profiles (Ctr *n* = 10, Ctr Stress *n* = 8, FVT Stress *n* = 9). Data presented as box plots display the minimum, first quartile, median, third quartile, and maximum.

We found an assortment of phages present in the FVT preparations, in most cases dominated by members of the class *Caudoviricetes* and the family *Microviridae*, although there are large portions of the viral population that are currently unassigned (Fig. 3c). There were 20 differentially abundant bacterial species that were altered by stress but restored when FVT was performed. Fifteen bacterial species were depleted by stress and protected by FVT, while 5 bacterial species were increased by stress and reduced by FVT (Fig. 3d and Extended Data Fig. 3a). Despite differences in individual species in the bacteriome, no changes were found in alpha- and beta-diversity metrics in the bacteriome or virome (Extended Data Fig. 4a–d), indicating that the bacteriome and virome did not undergo a large shift in community profile but rather, in low-abundance members and functionality.

The FVT oral gavage preparation underwent virome sequencing, allowing for the viral inputs to be characterized. Viral host pairs were predicted using a comprehensive collection of viral databases that cover the currently available bacteriophage community; however, these tools are still limited by the vast unknown portion of the bacteriophage virome^31^. Therefore, our bacteriophage sequencing data contain a small percentage of phage contigs that had a predictive host. Predicted hosts of the FVT inocula are reported at the phylum, order and family levels (Extended Data Fig. 3b–d). Phages targeting the phylum Deferribacterota and Bacteroidota (previously Bacteroidetes) were the most common, followed by Bacillota (previously Firmicutes) and to a lesser extent Pseudomonadota (previously Proteobacteria) and Halobacteriota. The most common phages targeted the orders Deferribacterales, Bacteroidales, Lachnospirales, Lactobacillales and Oscillospirales. Lastly, the most common phages targeted the families Mucispirillaceae, Bacteroidaceae, Erypsipelotrichaceae, Lachnospriaceae, Muribaculaceae, OLB10 and Streptococcaceae. However, the majority of the phage–host pairs in the murine microbiome are unknown.

**Extended Data Fig. 5:**
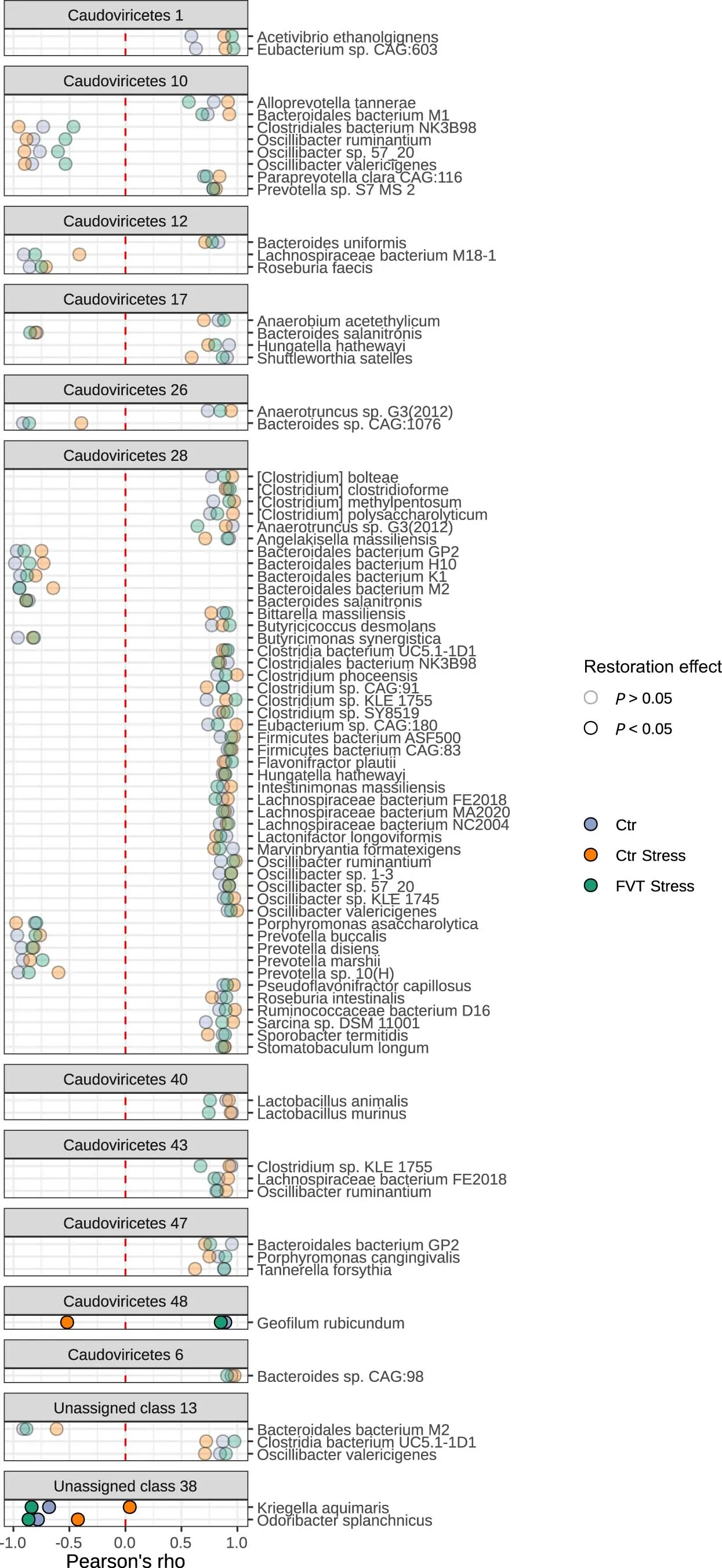
Phage-host network. Bacteriophage and bacterial abundance significant associations (*q* < 0.1). Phage-host pair associations that have a significant restoration effect are fully opaque (*P* < 0.05). Data was compared using two-sided linear models followed by Benjamini & Hochberg’s procedure for FDR and Pearson′s correlation coefficient to plot the phage-host pairs.

Phage–host pairs from the FVT preparation were then cross-referenced with the differentially abundant taxa (from faecal bacteriome sequencing data) that were affected by stress and restored by FVT. The identified viral taxa that overlapped with the differentially abundant bacterial species were depicted with the predicted phage hosts at the phylum level (Fig. 3e). At the phylum level, 18 of the 20 differentially abundant bacterial species were represented in the predicted phage–host pairs. There were 6 Pseudomonadota, 6 Bacillota, 3 Bacteroidota and 1 each of Verrucomicrobiota, Actinobacteriota and Cyanobacteria pairs. At the order level, there were 2 Burkholderiales, 1 Erysipelotrichales and 1 Bacteroidales pairs. At the family level, there was 1 Lachnospiraceae and 1 Erysipelotrichaeceae pair. This evidence supports bacteriome modulation by FVT, which in turn aided the restorative effects of the phage-derived treatment of stress. Given that phage infections in the gut are overwhelmingly temperate, the phage–host pair associations could be lytic or lysogenic in nature depending on environmental conditions, which can then have direct and/or indirect effects on bacterial host proliferation^9,34^. Phage–bacterial cascading interactions are shown as a correlational phage–host network plot (Extended Data Fig. 5). All phage–bacterial correlations depicted show significant association (*q* < 0.1), and specifically *Caudoviricetes* 48–*Geofilum rubicundum*, Unassigned class 38*–Kriegella aquimaris* and Unassigned class 38*–Odoribacter splanchnicus* show significant restoration effects following stress and FVT treatment (*P* < 0.05).

Across the differentially abundant Pseudomonadota taxa, the individual species increased in abundance following FVT compared with stress (*Advenella mimigardefordensis*, alpha proteobacterium AAP38, *Archangium gephyra*, *Nitrosomonas aestuarii*, *Paraburkholderia kururiensis* and *Sinorhizobium medicae*), indicating that FVT was a positive regulator. Five Bacillota taxa displayed increased abundance (*Bacillus aurantiacus*, *Bacillus campisalis*, *Butyrivibrio* sp. WCD3002, *Cohnella laeviribosi* and Erysipelotrichaeceae bacterium NK3D112). Conversely, the Bacteroidota species (*Paludibacter* sp. 47-17, *Pontibacter akesuensis*, *Thermonema rossianum*) decreased in abundance following FVT, as well as a single Bacillota species (*Paenibacillus* sp. 1-49) and the single Cyanobacteria species (*Oscillatoria* sp. PCC 10802), indicating that in these cases FVT was a negative regulator. These changes are attributable to stress modulation and either protection or reduction by the FVT treatment. Our data provide evidence that phage–host pairs are present and are potentially involved in the attenuation of stress-coping behaviours within our experimental parameters.

**Extended Data Fig. 6:**
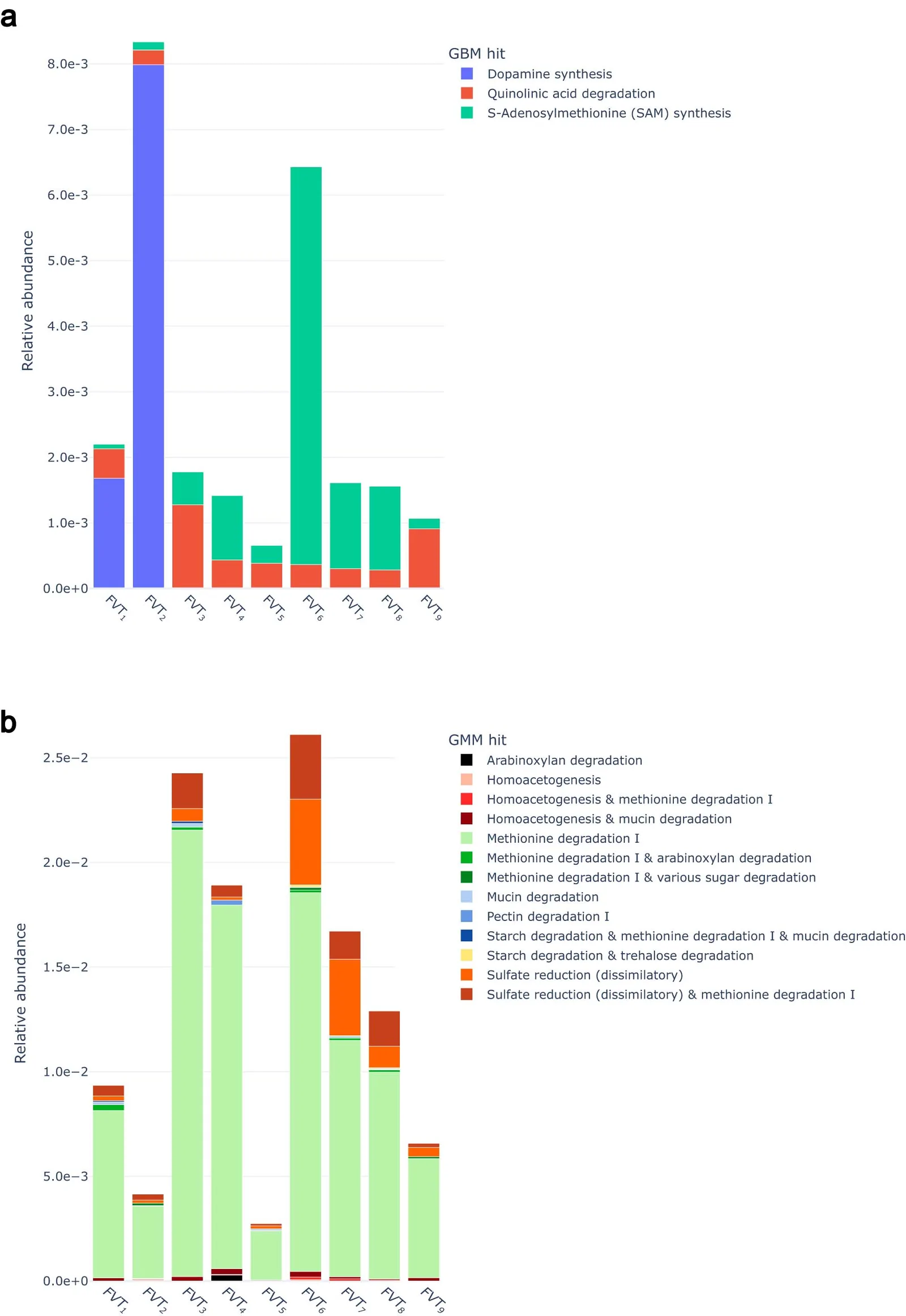
FVT inocula gut-brain and gut-metabolic modules. Faecal virome transplant (FVT) inocula were assessed for gut- brain module (GBM) and gut-metabolic module (GMM) functional carriage. (**a**) The GBMs found in the FVT inocula were dopamine synthesis, quinolinic acid degradation, and S- adenosylmethionine (SAM) synthesis. (**b**) The GMMs found in the FVT inocula were arabinoxylan degradation, homoacetogenesis, methionine degradation I, mucin degradation, pectin degradation I, starch degradation, and sulfate reduction (dissimilatory).

To assess the functional potential of the FVT inocula and bacteriome following the different treatments, gut–brain modules (GBM) and gut–metabolic modules (GMM) were characterized^35,36^. In the FVT inocula viral reads, there were three GBMs and ten GMMs identified (Extended Data Fig. 6a,b). Interestingly, the capacity for S-adenosylmethionine synthesis was found in each FVT inoculum and was significantly restored in the bacteriome GBM following stress and FVT. S-adenosylmethionine plays roles in a multitude of biochemical pathways both in the microbiota and across different tissues, including synthesis and metabolism of monoamine neurotransmitters^37–40^. We also assessed the restoration of GBM and GMM abundance by FVT in the bacteriome, but no other modules were found to be statistically significant following false discovery rate adjustment (Extended Data Tables 2 and 3).

### Stress-induced inflammation and immunity are protected by FVT

**Fig. 4:**
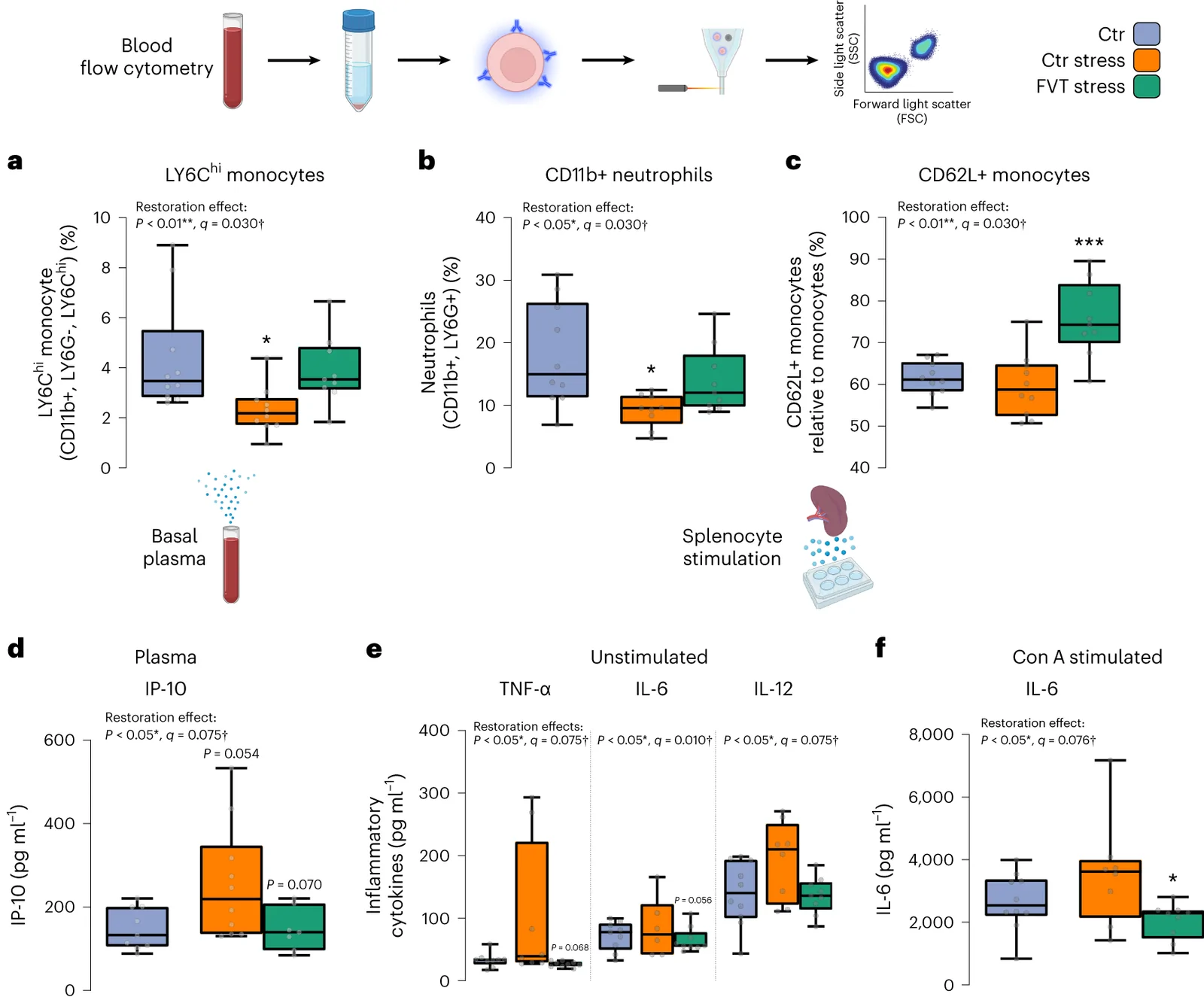
Effects of FVT on immune activation and inflammation. Blood immune cell populations were assessed by flow cytometry after 16 d of chronic social defeat. **a**, There was a restoration effect of relative LY6C^hi^ monocytes that were reduced by stress and recovered by FVT (restoration effect *P* < 0.01, BH-FDR-adjusted *P* value *q* = 0.030, *t*(26) = −2.867, Tukey’s pairwise comparisons *P* < 0.05, *P* = 0.106, respectively; Ctr *n* = 10, Ctr Stress *n* = 10, FVT Stress *n* = 9). **b**, There was a restoration effect of relative neutrophils that were reduced by stress and improved by FVT (*P* < 0.01, *q* = 0.030, *t*(25) = −2.788, *P* < 0.05, *P* = 0.231; Ctr *n* = 10, Ctr Stress *n* = 9, FVT Stress *n* = 9). **c**, There was a restoration effect of LY6C^hi^ monocytes carrying CD62-ligand that were not affected by stress but were strongly increased by FVT (*P* < 0.05, *q* = 0.030, *t*(26) = −3.18, *P* = 0.845, *P* < 0.001; Ctr *n* = 10, Ctr Stress *n* = 10, FVT Stress *n* = 9). **d**, Inflammatory cytokines were measured in plasma where there was a restoration effect for plasma IP-10 increased by stress and reduced by FVT (*P* < 0.05, *q* = 0.075, *t*(23) = 2.828, *P* = 0.054, *P* = 0.070; Ctr *n* = 9, Ctr Stress *n* = 10, FVT Stress *n* = 7). Inflammatory cytokines were also measured in unstimulated splenocyte culture supernatants and cultures that were stimulated with ConA or LPS. **e**,**f**, There were restoration effects for unstimulated splenocyte culture inflammatory cytokines of TNF-α (*P* < 0.05, *q* = 0.075, *t*(23) = 2.68, *P* = 0.086, *P* = 0.056; Ctr *n* = 9, Ctr Stress *n* = 8, FVT Stress *n* = 9), IL-12/IL-23p40 (*P* < 0.05, *q* = 0.075, *t*(24) = 2.634, *P* = 0.079, *P* = 0.068; Ctr *n* = 10, Ctr Stress *n* = 8, FVT Stress *n* = 9) and IL-6 (*P* < 0.05, *q* = 0.010, *t*(22) = 2.328, *P* = 0.162, *P* = 0.091; Ctr *n* = 9, Ctr Stress *n* = 6, FVT Stress *n* = 9) (**e**); and for IL-6 ConA stimulation of splenocytes (**f**) (*P* < 0.05, *q* = 0.076, *t*(24) = 2.517, *P* = 0.24, *P* < 0.05; Ctr *n* = 10, Ctr Stress *n* = 8, FVT Stress *n* = 9). In **a**–**f**, the restoration effect was measured using planned orthogonal contrast followed by Tukey’s post hoc test (two-sided pairwise comparisons of Ctr–Ctr Stress and Ctr Stress–FVT Stress) and BH-FDR adjustment. Data are presented in the following order: *P* = restoration effect measured by orthogonal contrast; *q* = BH-FDR-adjusted *P* value; *P* = Tukey’s post hoc test. Tukey’s pairwise post hoc *P* values were calculated for Ctr–Ctr Stress and Ctr Stress–FVT Stress. Data presented as boxplots display the minimum, first quartile, median, third quartile and maximum. Significant effects are denoted by: *P < 0.05, **P < 0.01, ***P < 0.001, †*q* < 0.2.

Stress-induced changes in immune cell populations and inflammatory cytokines provide evidence that stress-coping behaviour was impacted by prolonged immune activation. Peripheral immune cells and cytokines are known to be altered by stress and inflammation, which has been linked with increased anxiety-like behaviour in murine models^41–43^. Furthermore, both stress and corticosterone induce a redistribution of immune cells whereby myeloid cells migrate from reservoirs (for example, bone marrow, spleen) to facilitate surveillance and protection of immune-privileged tissues like the brain^44–46^. Characterizing the peripheral blood immunophenotype, we found that the populations of inflammatory monocytes and neutrophils were reduced by stress and improved by FVT (Fig. 4a,b). Interestingly, the monocytes present in the FVT recipient group strongly expressed the CD62-ligand (Fig. 4c), which is linked to lymphoid tissue migration shown in mice^47^. A chemokine measured in plasma (IP-10) and a series of proinflammatory cytokines in splenocyte cultures (TNF-α, IL-6 and IL-12) were elevated by stress and reduced by FVT, indicating a restoration effect of treatment (Fig. 4d–f and Extended Data Table 1b,c).

### FVT restores gene expression in the hippocampus and amygdala

Finally, to confirm that the FVT could also impact brain circuits underpinning anxiety and stress coping, we performed deep RNA sequencing in the hippocampus and amygdala of all animals. These stress-sensitive brain regions were selected on the basis of previous studies indicating that these regions were susceptible to stress and responsive to modulation by the microbiota^48–50^. The top enriched Gene Ontology (GO)^51^ terms from genes affected by stress and restored by FVT were selected to specifically target functions directly involved with stress, social behaviour, the virome and the microbiota–gut–brain axis. These GO terms consist of genes associated with fear response, immune system processes, neurotransmitter level modulation, postsynaptic neurotransmitter receptor functionality, stress response, social behaviour and viral processes.

**Fig. 5:**
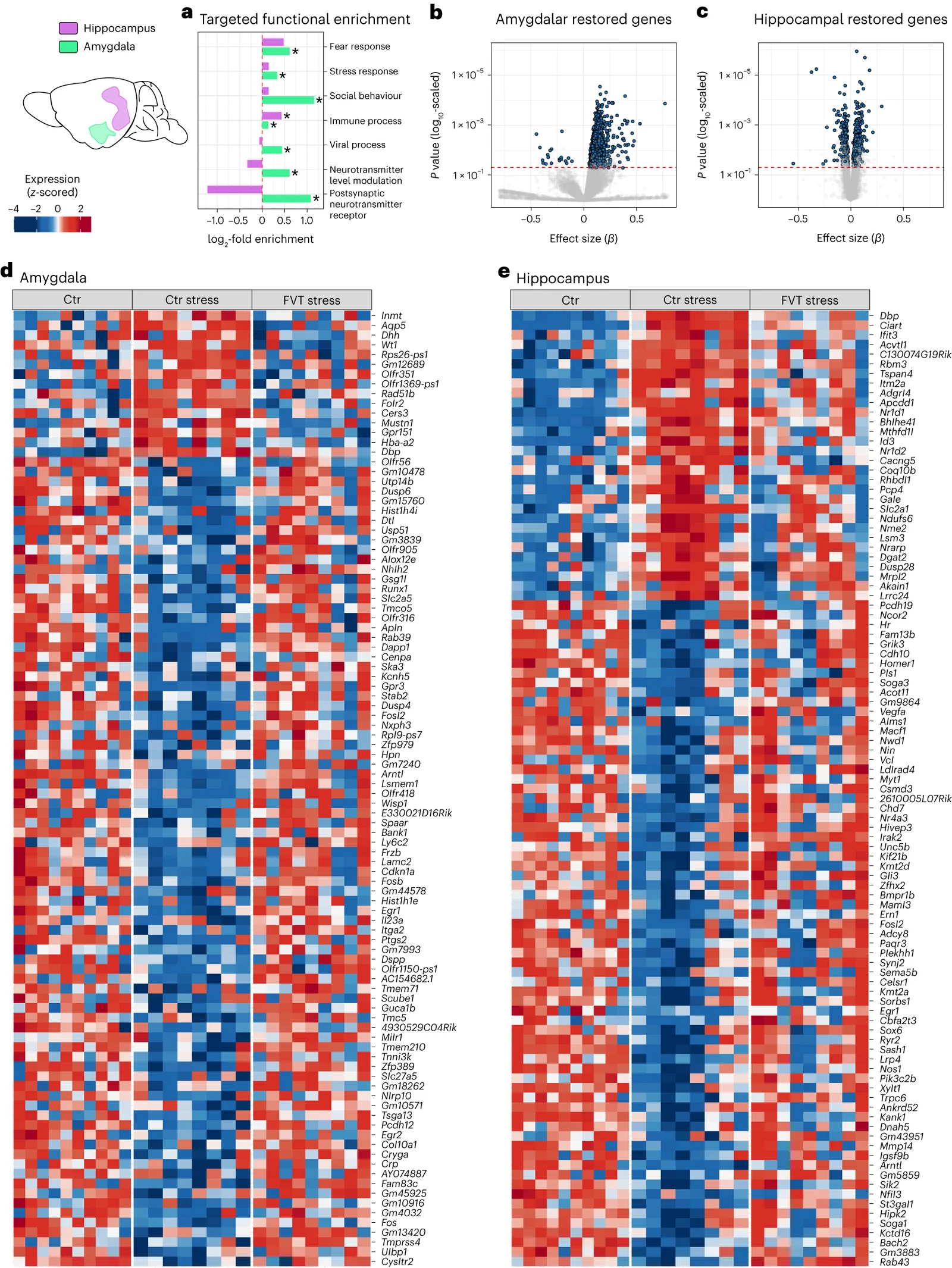
Amygdalar and hippocampal transcriptomic targeted gene ontology. Amygdala and hippocampus brain regions were selected for deep transcriptomic analysis for genes altered by stress and restored by FVT (restoration effect). **a**, Stress, social behaviour and microbiota–gut–brain axis targeted gene ontology of functional enrichment in genes restored in the hippocampus (purple) and the amygdala (green) (Ctr *n* = 10, Ctr Stress *n* = 8, FVT Stress *n* = 9; significant effects are denoted by *P < 0.001). **b**,**c**, Volcano plots showing the genes most significantly affected by stress and restored by FVT in the amygdala (**b**) (4,852 genes; 4,833 upregulated, 19 downregulated) and the hippocampus (**c**) (441 genes; 223 upregulated, 208 downregulated) (Ctr *n* = 10, Ctr Stress *n* = 8, FVT Stress *n* = 9). Genes (blue dots) to the right of ‘0’ are upregulated, while genes to the left of ‘0’ are downregulated following stress and FVT treatment. **d**,**e**, Heat plots showing the most significantly restored genes in the amygdala (**d**) and hippocampus (**e**). Differentially expressed gene ontology clusters and individual genes were assessed by linear models followed by Storey’s FDR.

In the hippocampus, immune system processes were significantly increased, while in the amygdala, all GO terms were significantly increased (Fig. 5a). This finding corresponds with the behaviour alterations observed via stress and FVT treatment and provides evidence that there is a differential regional response within the brain. Intriguingly, amygdalar genes that were affected by stress and restored by FVT (4,852) were largely upregulated (4,833 genes) rather than downregulated (19 genes; Fig. 5b,d). Hippocampal genes displayed an approximately equal number of genes positively and negatively modulated by stress and restored by FVT (441), with 233 genes being upregulated and 208 being downregulated (Fig. 5c,e). This indicates that there is a mechanism by which stress and FVT treatment impact neural gene expression that is particularly sensitive in the amygdala.

## Discussion

The gastrointestinal virome comprises an enormous and diverse population of mostly unclassified bacteriophages that can potentially modulate bacterial community structure and functionality^9,31^. Our study shows a permissive effect of stress on virome composition which can attenuate stress-related sequelae by targeting the bacteriome. First, we showed that chronic social stress affected both gut bacteriome and virome, in addition to eliciting stress-coping behaviour and deficits in immune function in mice. These findings correspond with previously described stress behaviour phenotypes^6^. Interestingly, stress increased the bacteriome and virome beta-diversity distance from controls in the faecal samples. These data highlight a potential link between stress-related behaviours and the gut bacteriome and virome. This led us to pursue the hypothesis that if the virome is permissive to the effects of stress, then we should be able to target the virome to attenuate the effects of stress.

We found that an autochthonous FVT could elicit protective effects against stress-coping behaviours through microbiota-mediated mechanisms. Following chronic social stress, FVT treatment improved social measures in the social interaction test and fully prevented the locomotor deficit observed in stressed animals. Anxiety-like behaviour measured by time spent and number of entries into the open arms of the elevated plus maze was similarly attenuated by FVT compared with the effects of stress. Moreover, there was a restoration effect of stress-induced elevations in circulating corticosterone following chronic stress and FVT treatment in response to acute stress. These findings indicate that there was a measurable stress behavioural response similar to previous reports from chronic social stress despite no differences observed in immobility time measured in the forced swim test^28,29^. Taken together, we show a reduction in anxiety-like behaviour, improved exploratory behaviour and attenuated stress coping in animals treated with FVT following stress.

The effects of the FVT on the bacteriome and virome were assessed through a combination of metagenomic and virome sequencing. These associated bacterial taxa have not previously been described in terms of chronic social stress or the microbiota–gut–brain axis at the species level. However, shared genera, which are more commonly characterized via 16S rRNA gene sequencing, provide some interesting context. Members of the *Bacillus* genus have been associated with social aggression prevention, alleviation of neuronal cell death and health improvement^52–54^. Moreover, a *Bacillus* species was found to attenuate high-fat diet-induced social deficits, anxiety-like behaviour, hippocampal oxidation stress and inflammation^55^. *Butyrivibrio* species are common producers of butyrate, which is a key short chain fatty acid product with diverse roles across the microbiota–gut–brain axis^56,57^. Erysipelotrichaceae, which had phage–host pair confirmation at every level assessed, was found to be differentially abundant following stress and FVT, and has been linked to inflammatory bowel diseases, metabolic disorders and enteral nutrition^58^. This family has been shown to be reduced in patients with recurrent or inflammatory conditions in Crohn’s disease^59,60^ and is a key taxon involved in bile salt metabolism^61^. However, there is also evidence that Erysipelotrichaceae is capable of driving inflammation during infection^62,63^. *Nitrosomonas* and *Nitrospira* species are involved in the nitrogen cycle in the gut and help to oxidize ammonia, which at elevated levels has been associated with reduced growth and immune dysfunction^64,65^, and can inhibit hippocampal synaptic transmission^66^. Interestingly, *Paenibacillus* produces a range of antimicrobials and insecticides. Since this microbe was increased following stress, it may indicate opportunistic and indirect pathobiont behaviour in that it has the potential to suppress other microbiota members^67^. There is evidence that members of the genus *Paraburkholderia* may become depleted following stress^68,69^. *Pontibacter*, also depleted by stress and restored by FVT, was previously associated with depression-like phenotypes^70^. In addition to these alterations in specific bacterial taxa, the FVT treatment has the capability to specifically change the dynamic activity of the microbiota. The FVT inocula were found to contain three gut–brain modules^35^ including quinolinic acid degradation, S-adenosylmethionine synthesis and dopamine synthesis. Furthermore, the FVT inocula also contained ten distinct gut–metabolic modules^36^ including arabinoxylan degradation, homoacetogenesis, methionine degradation I, mucin degradation, pectin degradation I, starch degradation and sulfate reduction (dissimilatory). These findings indicate that the faecal virome administered autochthonously during stress contains elements of both neuroactive and metabolic potential, which can play a role in the functional output of the microbiota.

The predicted phage–host pairs from the FVT solution overlapped with 90% of the cross-referenced differentially abundant bacterial species in response to stress and FVT at the phylum taxonomic level. However, only 10% similarly overlapped at the family level (Lachnospiraceae and Erysipelotrichaceae), given the vast yet undetermined portion of the gut virome and the focus of viral analysis tools on human rather than murine datasets^71,72^. Currently, bacteriophage databases are limited, as precise genetic determinants of phage host range are both highly specific and susceptible to mobile elements. For example, nominal amino acid changes in a tail fibre can alter receptor-binding domains of bacterial capsular structures and thereby change phage–host tropism^73,74^. Our data provide evidence that the FVT treatment contained phages that overlapped with bacterial hosts, indicating that there are phage–host pairs present; however, due to the aforementioned classification and sensitivity limitations, we cannot infer what the true percentages of the host–prey pairs were in our microbial communities. Two options potentially explain our relatively low phage–host pair numbers and lack of larger effects of FVT treatment on species differential abundance. First, the phage–host interactions in the gut are predominantly temperate with an inclination towards lysogeny, which did not result in community-wide alterations and fits with current gut bacteriophage models^9,34,75^. Second, it could be that the FVT exerted a controlling force over the microbial community through mutualistic interplay. This could occur by governing the growth of susceptible species in response to stress and selecting for species containing protective elements against predation^9,76^. It is classically perceived that phages are restricted to specifically infect bacteria and therefore do not elicit effects on mammalian cells. However, there is evidence that immune activation can occur following phage challenge^77,78^. Thus, the effects seen in our model are driven through FVT and presumably, phage modulation of the microbiota first, followed by the gut–brain axis, but direct effects of the phages can potentially occur. Furthermore, there is evidence that phage predation of susceptible bacteria can additionally have cascading effects on other species through interbacterial interactions^79^. Phage–host network correlational analysis of our dataset supports this dynamic cascade, as there are many phage–bacteria correlations that are not known phage–host pairs. Moreover, some of the phage–bacteria associations differentially coordinate between groups and may play a role in stress prevention and recovery.

In contrast to short-term models^44^, our chronic social stress model induced a reduction of peripheral inflammatory monocytes and neutrophils that coincide with increased levels of inflammatory cytokines, similar to previously seen chronic stress-induced immunosuppression effects^80^. Interestingly, there was a drastic increase in CD62L^+^ monocytes in circulation following FVT; this cell surface molecule plays an essential role in redistributing and guiding the migration of monocytes into inflamed tissue in stress, suggesting that peripheral monocytes were actively being recruited^45^. Plasma IP-10 underwent a significant restoration effect following an elevation by stress and reduction by FVT. This cytokine belongs to the CXC chemokine family and has been shown to be induced by TLR-9 stimulation and in turn to stimulate monocytes, T-cell migration and other immune cells to promote viral clearance^77,81,82^. The spleen is an immune cell reservoir and splenocyte culture allows ex vivo testing to assess cytokine production of the immune cell population including monocytes, macrophages, T and B lymphocytes, and dendritic cells^46^. Similar restoration effects following stress and FVT were seen in unstimulated splenocyte production of inflammatory cytokines TNF-α, IL-6 and IL-12. IL-6 was the only cytokine reduced by FVT following Con A splenocyte stimulation, further indicating that the FVT treatment was able to reduce antigenicity hyper-response following stress. Here, our collective cytokine data suggest that the FVT stress group did not show signs of immune activation, viral detection or active infection. Immune cell signalling is a key mechanism by which the gut microbiome influences the brain, including in stress^41^, and it is plausible that the ability of FVT to mitigate the stress response is driven through immune-mediated pathways^83^, although more mechanistic research would need to be done to fully elucidate specific microbe–immune–brain pathways involved.

Transcriptomics of the hippocampal and amygdalar regions of the brain revealed many genes that were stress and FVT sensitive. Both the amygdala and hippocampus are brain regions highly involved in coordinating stress response and are sensitive to the gut^48,84,85^. The amygdala has been shown previously by our group to be particularly sensitive to anxiety, social behaviour and microbiota interactions^84,86^. We observed that the expressions of the vast majority (99.6%) of amygdalar genes were reduced by stress and restored by FVT, while in the hippocampus, gene expression levels were more evenly distributed (52.8% were upregulated following stress and FVT). In the hippocampus and amygdala, GO immune terms were restored; in the amygdala, genes associated with endogenous viral processes were additionally increased. These viral processes include viral multi-organism interactions such as host cell infection, viral genome replication and/or assembly of virus particle progeny. Since the brain is an immune-privileged organ protected from microbial infection, the viral processes are probably endogenous retroviral assemblages historically incorporated into the genome of the host. In other contexts, these sequences of retroviral origin have been associated with homoeostatic and inflammatory responses promoted by the microbiota^87^. Furthermore, amygdalar GO behavioural terms displaying genes associated with social, stress and fear behaviours were restored. Lastly, neurotransmitter and postsynaptic membrane neurotransmitter receptor levels were additionally restored in the amygdala.

It is worth noting that one of the challenges facing current phage research is that phage sequence databases are primarily made up of industrially relevant, pathogen-associated and/or cultivatable phages. Given that the majority of sequenced samples in our study are unknown murine phages, our ability to classify all the viral sequences found is limited. Another limitation is that single-stranded (ss)DNA and RNA viruses are frequently not captured in sequencing analyses. To account for this, we used a reverse transcription step to convert ssDNA/RNA to complementary (c)DNA, which is double-stranded and therefore can be incorporated in the library preparation. No viruses were identified taxonomically to belong to viral taxa that have RNA genomes in the initial virome sequencing (characterizing Ctr vs Stress). However, in the second virome analysis (the FVT study), putative signals belonging to taxa with RNA genomes were observed (for example, ssRNA viruses *Astroviridae* and *Fiersviridae*, formerly *Leviviridae*) albeit at very low levels in comparison with those for viruses with DNA genomes. Putative signals corresponding to ssDNA viral taxa were also identified in the FVT viromes (for example, *Circoviridae*, *Genomoviridae*, *Microviridae*, *Parvoviridae*). It should also be noted that a considerable number of viral contigs within both datasets could not be classified to belong to known viral taxa (that is, ‘unassigned’), and while this does not mean that these are additional RNA/ssDNA viruses, it also does not exclude this as a possibility.

By characterizing the FVT inocula using epifluorescence microscopy and shotgun sequencing, we show that the purified FVT inocula comprised virus-like particles and viral reads, but as we primarily selected phages on the basis of size, it is comprehensible that the smallest bacteria at the extremes of life might have also been transferred. However, the purification steps used herein specifically isolated and concentrated viruses that were between 100 kDa (∼3.05 nm) and 45 µm using centrifugation, extensive filtering and dialysis, which would remove larger microbes and smaller metabolites, proteins and the extracellular milieu. The next consideration is the temperate life cycle, which has the capability to elicit both lytic and lysogenic pathways depending on environmental conditions and is the predominant phage life cycle type in the gut^9^. This makes it challenging to ascertain whether phages are impacting their bacterial hosts via direct lytic action or through the multimodal optionality of lysogeny. We can infer that since the FVT was derived from excreted faeces, the phages were predominantly temperate, as it is theorized that phages in the top mucin layer primarily follow ‘piggyback the winner’ dynamics whereby incorporated phages multiply as a result of bacterial division and increase in abundance together^88^. Furthermore, the FVT would also have a bias towards the free viral fraction since incorporated plasmids and lysogenized viral nucleic acid motifs are not readily collected in the sample preparation. This would mean that there are elements of the original donor virome that would not be transferred. Lastly, administration of the FVT by oral gavage is an inherently stressful technique which we balanced by also performing a gavage of buffer to the control groups, but this could potentially impact outcomes as an additional stress-related measure.

FVT as a technique is still in its infancy but there are increasing data supporting its efficacy in disease models^11,14,89–91^ and it is burgeoning as an alternative microbiota-based treatment^91–93^. Recent evidence suggests that it is possible to inactivate different strata of the FVT^90^, which may increase specificity of treatment by targeting specific hosts. By using this approach, it is possible to further dissect what groups are driving phage–bacteria–host interactions. Furthermore, it is also necessary to assess what effects a fully inactivated FVT would have on the microbiota and mammalian host. Alternatively, as the role and function of specific microbial taxa are uncovered, phage cocktails targeting pathobionts can be assembled and delivered to ameliorate associated maladies. Our goal for this study was to determine whether the gut virome could elicit a therapeutic effect to prevent stress-related behaviours and then to perform a deep phenotyping analysis to describe the effects in the context of the microbiota–gut–brain axis. Under the specific conditions of our study, we observed protective effects of an autochthonous FVT against stress; however, the specific agents that drive these effects and the targets they interact with are yet unknown and will require future studies to unravel this nuanced interplay. There are yet many intriguing possibilities and directions for future research including whether an inactivated FVT can elicit effects, characterizing how an FVT affects the host in lieu of a host microbiota (such as with germ-free or gnotobiotic models), whether an FVT from a stressed animal could elicit a stressed phenotype and whether specific phages can have anti-stress potential.

In conclusion, we show that the virome plays a permissive role to the microbiota–gut–brain axis response to stress. This response can be targeted through the virome (that is, FVT) to ameliorate the effects of stress on the host at a multitude of levels. Bacteriophage treatment demonstrates a utility for harnessing individual strata within the microbiota–gut–brain axis to protect against stress. As gut phage research progresses, characterization of the dynamic interplay between phage and host within the microbiota will unlock even greater potential to promote gut and brain health.

## Methods

### Animals

In the first study, 20 male C57Bl/6J mice (*n* = 10 per group) and 20 male CD1 non-experimental resident aggressor mice were used; in the second study, 30 male C57Bl/6J mice (*n* = 10 per group) and 50 male CD1 mice were used and housed within the Biological Services Unit, University College Cork. Mice were received at 8 weeks of age from Envigo (UK) and randomly assigned to groups. Animals were habituated for 1 week after which the ‘stress’ groups were single housed while the control groups remained group housed. Food and water were provided ad libitum throughout the study. The holding room was under a 12 h light/dark cycle (light cycle began at 07:00), with a temperature of 21 ± 1 °C and humidity of 55 ± 10%. Body weight was monitored twice per week. To provide an adequate sample size to detect changes; a priori power analysis was performed using the software G*Power (v.3.1) considering a power of 80% (1-beta-error) and a 0.05 alpha-error. Investigators were not blinded during allocation of experiments; however, analyses were conducted in a manner blinded to the experimenter. All procedures were conducted with approval from the Animal Experimentation Ethics Committee (AEEC) at University College Cork and the Health Products Regulatory Authority (HPRA) in accordance with the recommendations of the European Directive 2010/63/EU.

### Experimental timeline and behaviour testing

There were two groups, Stress and Ctr, used in the first experiment to test whether chronic social stress changed the bacteriome and virome. Animals (assigned randomly) in the Stress group underwent chronic psychosocial defeat stress which consisted of a semi-randomized sequence of social defeat and overcrowding stressors over a period of 20 d (see Fig. 1a). Behavioural tests were performed between 9:00 and 12:00 as follows: (1) Day 21, social interaction test; (2) Day 22, forced swim test. At the end of the study, animals were euthanized on Day 23 by decapitation and tissues were collected for subsequent analyses.

Animals were randomly assigned to one of three groups used in the second experiment: Ctr, Ctr Stress and FVT Stress. At the start of chronic social stress, animals additionally received treatment by oral gavage; Ctr and Ctr Stress groups received vehicle saline magnesium (SM) buffer (50 mM Tris-HCl, 100 mM NaCl, 8.5 mM MgSO_4_; pH 7.5), and the FVT stress group received the purified autochthonous faecal virome from the pre-stress faecal collection. Animals in the ‘Stress’ group underwent chronic social defeat stress 19 times over 22 d (animals were not stressed on the three behaviour testing days). Control animals did not receive stress but were handled evenly by researchers at the same time. Behavioural tests were performed between 9:00 and 12:00 as follows: (1) Day 11, social interaction test; (2) Day 15, elevated plus maze; (3) Day 19, forced swim test (see Fig. 2a). At the conclusion of the study, animals were euthanized on Day 23 by decapitation and tissues were collected for subsequent analyses.

### Faecal virome purification

Faecal samples were collected in a PBS-glycerol (20%) solution, frozen (−80 °C) and later used to generate phage-rich material used for FVT oral gavage. Approximately 2.5 g of faecal pellets were resuspended in 10 ml filter sterilized SM buffer and 2 ml 1 M NaHCO_3_ (to reduce stomach acidity). The faecal slurry was vigorously vortexed and then centrifuged at 5,000 *g* for 20 min. The supernatant was then filter sterilized (0.45 μm filter) before undergoing overnight dialysis (SpectraPor Float-A-Lyzer Dialysis Device G2, 100 kDa, Repligen).

### Chronic psychosocial stress

In the first experiment, mice underwent 20 d of chronic psychosocial stress, which comprised social defeat stress and/or overcrowding (see Fig. 1a for experimental timeline)^26^. Experimental mice were stressed over a consecutive 20-d period in an unpredictable social defeat/overcrowding schedule. Social defeat sessions (described below) included exposure to an aggressive CD1 mouse and attack or submissive posture. Overcrowding sessions included co-housing for 24 h in a standard holding cage with *n* = 10 per cage.

### Chronic social defeat stress

In the second experiment, chronic social defeat stress was carried out daily for 19 consecutive days (excluding behaviour test days; see Fig. 2a for experimental timeline)^94^. Chronic social defeat stress was selected to eliminate potential coprophagy events that may occur during the overcrowding portion of the chronic psychosocial stress paradigm that may confound the effects of FVT treatment. CD1 mice were screened for aggression over 2 d before social defeat sessions. CD1 mice with the shortest attack latencies were selected as the aggressors to be used in subsequent social defeats. For each social defeat session, a CD1 mouse novel to the experimental mouse was used. The session would involve an initial exposure to the CD1 mouse in a clean cage with fresh bedding to reduce coprophagy. The session would last until the first attack, expression of submissive posturing by the experimental mouse, or until 5 min had passed. The latency to attack or submission was recorded. The mice were then separated by a clear Plexiglas perforated wall that allowed non-physical contact for 2 h. After 2 h, the separator was removed, a second defeat session was conducted, then mice were returned to their home cages. Social defeat sessions were carried out in the afternoon during the light cycle. Control mice were handled an equal amount daily in lieu of social defeat.

### Social interaction test

For study one, animals underwent the social interaction test^95^ after 20 d of chronic psychosocial stress; for study two, animals were tested after 10 consecutive days of chronic social defeat stress. Experimental animals were placed in a rectangular plastic box (31 × 39 cm^2^) containing an empty wire mesh cage (9.5 × 7.5 cm^2^) and allowed to freely explore for 2.5 min, then the mouse was removed and placed in its home cage for 1 min while a novel CD1 mouse was placed into the wire mesh cage. The experimental animal was then returned to the social interaction box and allowed to freely explore for 2.5 min while the CD1 mouse was in the wire mesh cage. At the conclusion of the test, the mice were returned to their home cages and the box and wire mesh cage were cleaned with 70% ethanol. Trials were recorded with a ceiling-mounted camera and evaluated using Ethovision 11.5 (Noldus). Social interaction and corner ratios were calculated by taking the ratio of the time spent in the interaction zone (area around the mesh cage) or in the corner (the two furthest points from the mesh cage) during the habituation versus when the CD1 was present.

### Elevated plus maze test

The elevated plus maze test was used to assess anxiety-like behaviour and was conducted following ref. ^28^. The maze apparatus was elevated 1 m above ground and consisted of a grey plus (+)-shaped maze with two opposing open arms and closed arms (50 × 5 cm arm length, 15 cm closed wall height, 1 cm open wall height). Experiments and habituation were conducted in red light (5 lux). Mice were habituated to the room for 1 h before the test, then allowed to explore the maze for 5 min. The maze was cleaned with 70% ethanol after each animal trial. Experiments were recorded with a ceiling-mounted camera and videos were scored blind for time spent in open arms and open arm entries (entrance defined as all paws in arm).

### Forced swim test

The forced swim test was used to assess depressive-like stress-coping behaviour and was conducted according to ref. ^28^. A glass cylinder (21 cm diameter) was filled with 24 ± 1 °C water to a height of 15 cm; water was emptied and replaced after each individual run. Experimental animals were placed in the water for 6 min while being recorded by a ceiling-mounted camera. After each test, animals were gently dried off with a towel and returned to their home cage. The last 4 min of the 6 min test was used to manually score immobility behaviour from the video recorded by a researcher blinded to the experimental groups. Mice were judged to be immobile when only making movements necessary to keep their head above water.

### Repeated blood sampling for stress and immune measures

Plasma from each animal was sampled by a tail tip 5 min before the forced swim test, then 15, 45 and 90 min after the start of the test. The tail was securely restrained and a diagonal incision was made ∼1–2 mm from the tip of the tail. Approximately 40 μl of whole blood was collected in an EDTA-lined capillary tube (Fisher Scientific, 749311) per timepoint. Samples were deposited in an Eppendorf and centrifuged for 10 min at 3,500 *g* at 4 °C. Plasma was collected and stored at −80 °C for later corticosterone quantification. The remaining blood cells were used for immune flow cytometry analysis.

### Corticosterone stress response timeline

Samples were analysed in duplicate in a single assay using 10 μl plasma per sample. The threshold of detection was <32 pg ml^−1^, with coefficient of variation limit of 20% and concentration units expressed in ng ml^−1^. Light absorbance was read with a multimode plate reader (Synergy HT, BioTek Instruments) at 405 nm.

### Flow cytometry

**Extended Data Fig. 7:**
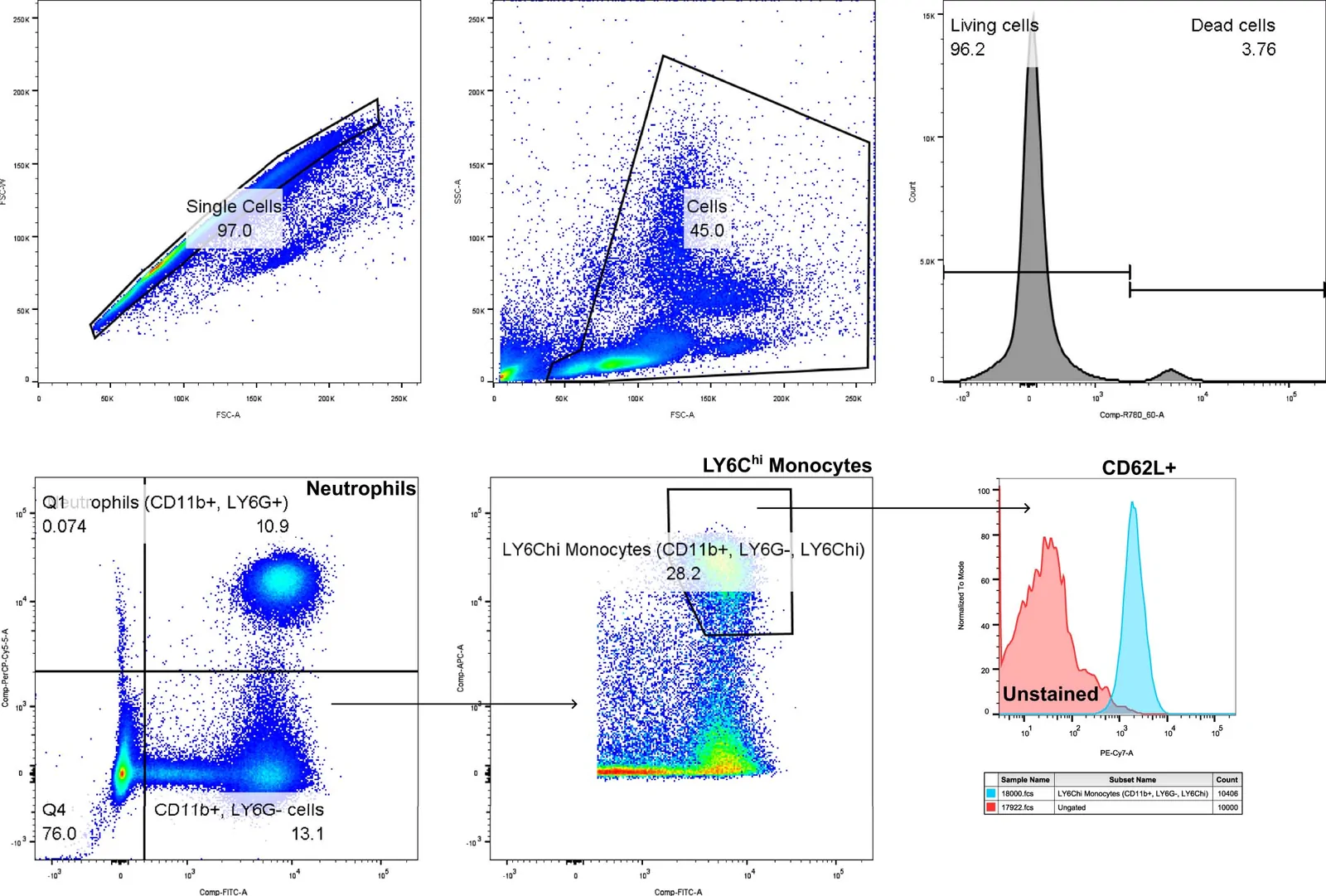
Immune cell flow cytometry gating strategy. A panel of whole blood markers was used to assess circulating immune cell populations on the FACSCelesta. This approach enabled initial gating for singlets, followed by the selection of the peripheral blood mononuclear cells, and live cells (using FVS780). Neutrophils were selected based on LY6G+ (PerCP- Vio700-conjugated) and CD11b+ (FITC-conjugated). Monocytes were selected as being Ly6G−, CD11b+ and LY6Chi (APC-conjugated), after which CD62L+ cells (Pe-Cy7- conjugated) were quantified.

Flow cytometry was performed on blood samples collected before and 45 min after forced swim test, and samples were processed on the same day. Staining, gating and analysis were performed according to the following methods^94^ and a gating strategy is provided in Extended Data Fig. 7. Blood was resuspended in 10 ml of red blood cell lysis buffer for 3 min (15.5 mM NH_4_Cl, 1.2 mM NaHCO_3_, 0.01 mM tetrasodium EDTA, diluted in deionized water). Samples were then centrifuged (1,500 *g*, 5 min) and resuspended in 45 μl staining buffer (autoMACS rinsing solution, Miltenyi 130-091-222, supplemented with MACS BSA stock solution, Miltenyi 130-091-376) for the staining procedure. Then, 5 μl of FcR blocking reagent (Miltenyi, 130-092-575) was added to each sample. Next, samples were incubated with a mix of antibodies at a 2:71 dilution (CD11b+ Miltenyi, 130-113-234; LY6G Miltenyi, 130-117-500; LY6C Miltenyi, 130-111-917; CD62L BioLegend, 104418; MHC-II Biolegend, 107632; CD4 Biolegend, 100548; CD44 BD Biosciences, 563736; CD69 Miltenyi, 130-115-575) and incubated for 30 min on ice. Samples were then fixed in 4% paraformaldehyde (PFA) for 30 min on ice. Fixed samples were resuspended in staining buffer and analysed the following day on the FACSCelesta flow cytometer (BD Biosciences) with FACSDiva (v.8.0, BD Biosciences) and CellQuest Pro (v.5.2, BD Biosciences). Data were analysed using FlowJo (v.10). Cells were gated on the basis of FSC-height and SSC-height; subsequently, LY6C^hi^ monocytes (CD11b^+^, LY6C^hi^), neutrophils (CD11b^+^, LY6C^+^, LY6G^+^) and T helper cells (CD11b^−^, LY6G^−^, CD4^+^) were selected. Next, the proportions of LY6C^hi^ monocytes and neutrophils that contained CD62L or MHC-II were assessed relative to the respective parent population. Then, the proportions of T helper cells that were either CD69^+^ or CD44^hi^ were similarly assessed relative to the total parent cell population.

### Splenocyte stimulations

Spleens were collected during tissue collection, trimmed of fat and placed in RPMI medium (R8758, supplemented with 10% fetal bovine serum (F7524, Sigma) and 1% penicillin-streptomycin-glutamine 100x (10378-016, Biosciences)). They were then treated with red blood cell lysis buffer (R7757, Sigma), passed through a 70-μm filter and then resuspended in fresh supplemented RPMI medium. Next, 4 ml of splenocytes were seeded in 6-well plates at a concentration of 16 × 10^6^ cells per well. After a 2.5 h rest period, cells were stimulated with lipopolysaccharide (2 μg ml^−1^, tlrl-eblps, Invivogen) and concanavalin A (2.5 μg ml^−1^, C5275, Sigma) for 24 h to induce ceiling antigenicity. Then, supernatant was collected and used for inflammatory cytokine analysis.

### Inflammatory cytokine analysis

Cytokine levels from plasma and splenocyte supernatant samples were collected during euthanasia and quantified using the V-PLEX Proinflammatory Panel 1 mouse kit (MSD, K15048D), measuring IFN-y, IL-6, IL-12/IL-23p40, TNF-a and IP-10 on the MESO QuickPlex SQ 120 SECTOR Imager 2400 (MSD). Cytokine quantification was done according to manufacturer guidelines. Samples below the detection limit of each assay were excluded.

### Faecal bacteriome collection and sequencing

In the first experiment, the QIAamp DNA stool mini kit (Qiagen) was used to extract bacterial DNA using the manufacturer’s handbook (2nd edn 2012) ‘Isolation of DNA from Stool for Pathogen Detection’. Samples were prepared for 16S sequencing using the Nextera XT DNA library prep kit (Illumina), as described in the Illumina 16S library preparation workflow. 16S bacterial rRNA gene was amplified using primers targeting the V3–V4 hypervariable region (Forward: 5′TCGTCGGCAGCGTCAGATGTGTATAAGAGACAGCCTACGGGNGGCWGCAG; Reverse: 5′GTCTCGTGGGCTCGGAGATGTGTATAAGAGACAGGACTACHVGGGTATCTAATCC) (Sigma Aldrich). The Illumina V3–V4 primers were selected for their high coverage (94.5% bacteria) while remaining in the amplicon size necessary for sequencing^96^. 16S rRNA amplicons were sequenced on the Illumina MiSeq platform (Teagasc, Moorepark, Ireland).

In the second experiment, The QIAamp Fast DNA stool mini kit (Qiagen) was used to extract DNA from faecal pellets. This was performed following manufacturer’s guidelines. Before extraction, however, 20 μl of the ZymobIOMICs spike-in control 1 (Zymo Research) was added to aid in calculating absolute abundances. DNA concentrations were then measured using the Qubit High Sensitivity assay (Thermo Fisher Scientific) before adjustment to 0.2 ng μl^−1^ for Nextera XT library preparation (Illumina). Library quality was assessed with the 4200 Agilent Tapestation (Agilent Technologies) using the High Sensitivity D1000 ScreenTape assay (Agilent Technologies) before whole metagenomic sequencing was performed on an Illumina Novaseq platform using 2 × 150 nt paired-end chemistry (Genewiz).

### Faecal virome collection and sequencing

Faecal viromes in the initial experiment were processed using a verified protocol^34,97^. Briefly, the samples from control and stress groups were pooled (*n* = 3–4 per pool) to create 1–2 g faecal samples. Pooled samples were resuspended in 10 ml SM buffer, vortexed for 5 min, cooled for 5 min on ice and centrifuged at 5,000 *g* for 10 min at 4 °C to remove large particulate and bacterial cells, then supernatants were transferred to new tubes and centrifugation was repeated. Supernatant was filtered twice through a 0.45-µm-pore ceramic filter. Following further refinement and treatment with proteinase K and phage lysis buffer, sample DNA was extracted using the phenol-chloroform method. After DNA extraction, samples were processed for shotgun metagenomic sequencing using the Nextera XT DNA library prep kit (Illumina). Virome metagenomic sequencing was performed on the Illumina MiSeq platform (Teagasc, Moorepark, Ireland).

In the second experiment, an updated protocol described in ref. ^98^ was used. For FVT gavage samples, 400 µl were processed, and to enable comparisons of viral load across samples, 10 µl of 10^8^ plaque-forming units per ml of lactococcal phage Q33 were added to each sample. For faecal samples, a single faecal sample from each mouse was pooled per group in 650 µl of SM buffer. Fresh 0.5 M dithiothreitol was prepared in 1 ml of SM buffer. A volume of 16 µl of the dithiothreitol stock was added to samples to achieve a final concentration of 12 mM and samples were incubated at 37 °C for 30 min. Centrifugation at 4,000 *g* for 30 min at room temperature was used to pellet debris and bacterial cells. Subsequently, 400 µl of liquid was removed and treated with 40 µl of DNase/RNase buffer (50 mM MgCl_2_, 10 mM CaCl_2_), 12 µl (24 U) of Turbo DNase (Thermo Fisher) and 4 µl (40 U) of Fermentas RNase I (Fisher Scientific) at 37 °C for 1 h. Nucleases were inactivated by incubating at 65 °C for 10 min. With free nucleic acids now removed, virus-like particles (VLPs) were lysed using the QIAgen blood and tissue kit following manufacturer guidelines. However, an elution volume of 30 µl of Qiagen AE elution buffer (10 mM Tris-HCl, 0.5 mM EDTA, pH 9.0) was used to increase the final concentration of nucleic acid obtained.

A reverse transcription reaction was performed using the SuperScript IV First Strand Synthesis System (Invitrogen/Thermo Fisher Scientific) with 11 µl of purified VLP nucleic acid sample and random hexamer oligonucleotides according to manufacturer protocol. The concentration of DNA purified using a DNeasy blood and tissue kit (Qiagen) was determined using the Qubit dsDNA HS kit and the Qubit 3 fluorometer (Invitrogen/Thermo Fisher Scientific). DNA/cDNA yields varied between 0.05 and 29 ng µl^−1^, with some samples being below the detection limit. Before library preparation, 22 µl of each sample underwent reverse transcription using the Superscript IV kit (Invitrogen/Thermo Fisher Scientific) according to manufacturer protocol to include any RNA viruses in the analysis.

Subsequently, samples were sonicated following adjustment of the volume to 52.5 µl with low-EDTA TE buffer. Shearing of unamplified DNA/cDNA mixture was performed on an M220 Focused-Ultrasonicator (Covaris) with the following settings: peak power of 50 W, duty factor of 20%, 200 cycles per burst, total duration of 35 s. Samples were then concentrated using the Genomic DNA Clean and Concentrator-10 kit (Zymo Research) and eluted in 16 µl aliquots. Library preparation was then carried out using the Accel-NGS 1S Plus kit (Swift Biosciences) according to manufacturer instructions. However, a final and additional bead clean-up step of 0.8 DNA/AMPure beads v/v ratio was included. Library QC to determine fragment length distribution and quantitation was performed with the 4200 Agilent Tapestation (Agilent Technologies) using the High Sensitivity D1000 ScreenTape assay (Agilent Technologies) and Invitrogen Qubit. The normalized pooled library was then sequenced using 2 × 150 nt paired-end sequencing chemistry on the Illumina HiSeq platform at Genewiz.

### Analysis of bacteriome sequencing data

We performed quality checks on raw sequences from all faecal samples using the FastQC programme with a quality score of 30 as a threshold. Shotgun metagenomic sequencing data were then cleaned and host genome sequences were filtered using Bowtie2 via the Kneaddata wrapper programme with following parameters: ILLUMINACLIP:/NexteraPE-PE.fa:2:30:10, SLIDINGWINDOW:5:25, MINLEN:60, LEADING:3, TRAILING:3. Reads were aligned to the Web of Life database using Bowtie2, and taxonomic and functional profiling of the microbial community was performed using Woltka. Next, the uniref90-based gene abundance matrix was further collapsed using KEGG Orthology (KO) term mapping via the ‘woltka tools collapse’ function provided within Woltka. Woltka SOP is available online (https://github.com/qiyunzhu/woltka/blob/master/doc/wolsop.sh). GBMs^35^ and GMMs^36^ were calculated using the R version of the Gomixer tool.

### Analysis of gavage virome sequencing data

Virome reads were processed to remove adaptors, trim low-quality bases, and filter reads for quality and length using fastp (v.0.23.2)^99^ with the flags ‘-length_required 60–detect_adapter_for_pe –cut_front –cut_tail –cut_window_size 4 –cut_mean_quality 20–trim_front1 10’. Eukaryotic reads were then identified and removed by mapping reads to the *Mus musculus* and *Homo sapiens* genomes (GCF_000001635.27_GRCm39 and GCF_009914755.1_T2T-CHM13v2.0, respectively) using Kraken (v.2.1.2)^100^. At each step, read quality was assessed using FastQC (v.0.11.9)^101^.

Reads passing the trimming and filtering steps were then assembled on a per-sample basis using MEGAHIT (v.1.2.9)^102^ with default parameters. The resulting contigs from all samples were then pooled and filtered to retain only those contigs ≥3 Kbp in length. Redundant contigs were then removed following an all-vs-all BLASTn (v.2.12.0+) comparison (*e*-value cut-off of ≤1 × 10^*−*20^) where the shortest contig was removed for all pairs of a hit for contigs that shared ≥90% identity over ≥90% of the shortest length of the contig.

Putative viral contigs from the non-redundant contig pool were then identified using the following criteria: first, contigs were compared to the viral nucleotide sequences within the National Center for Biotechnology Information (NCBI) RefSeq database release 211, the Gut Virome Database^103^, the Joint Genome Institute (JGI) IMG/VR v.3 (release #5; 12-10-2020)^104^ and our in-house crAss-like bacteriophage genome database using BLASTn (v.2.12.0+) with an *e*-value cut-off of ≤1 × 10^−10^. For these nucleotide comparisons, any contig found to have ≥85% of its length covered by any combination of hits with at least 50% identity to sequences within these databases was deemed as a potential viral sequence. As phages are particularly heterogeneously distributed among microbiomes if a feature is absent (or unobserved) in approximately half of samples, inclusion in subsequent analysis greatly increases the risk of spurious associations^105^. Potential viral contigs were also identified on the basis of protein homology. For this, open reading frames (ORFs) were called within each contig with Prodigal (v.2.6.3)^106^ using the ‘-meta’ option, with the resulting ORFs compared to the NCBI RefSeq viral protein database (release 211) using BLASTp (v.2.12.0+; *e*-value cut-off of ≤1 × 10^−10^). ORFs were also compared to the hidden Markov models (HMMs) of the Prokaryotic virus Remote Homologous Groups (PHROGs; release v.3) database^107^ and the JGI’s inovirus protein families (iPFs) database^108^ using hmmscan (HMMER v.3.3.2; *e*-value cut-off of ≤1 × 10^−5^). Contigs were denoted as potentially viral from the results of protein comparisons if the contig encodes for at least 2 ORFs that were hit by any combination of the protein comparisons where this accounted for at least 50% of the total of the contig ORF count. Two stand-alone viral identification tools were also used to identify putative viral contigs from the contig pool, namely VirSorter2 (v.2.2.3)^109^ (with ‘–include-groupsdsDNAphage,RNA,ssDNA’) and DeepVirFinder (v.1.0)^110^ (with default paramaters). Finally, any circular contigs identified by the ViRal and Circular content from metAgenomes tool (VRCA; https://github.com/alexcritschristoph/VRCA) were also included in the list of potentially viral contigs.

Contigs that met any of these viral identification criteria were then examined for the presence of rRNA genes using barrnap v.0.9 (https://github.com/tseemann/barrnap) with default parameters, with any contigs found to contain rRNA hits then removed from the list of potential viral contigs. Finally, the potential viral contigs were then examined for quality using CheckV v.1.0.1 and its sequence database (v.1.2)^111^. In cases where a contig was identified as proviral by CheckV, the contig sequence was refined to that within the viral start and end positions suggested by CheckV for that contig. As this refinement altered some of the viral contig lengths, contigs where again re-filtered to remove contigs ≤3 Kbp in length.

From the list of viral contigs, the assembled contig corresponding to the lactococcal phage Q33 used as an internal viral standard was identified by BLASTn of the phage Q33 genome (GenBank accession: JX564242.1) and the corresponding contig replaced with the reference genome sequence for Q33. Viral contigs were then clustered into viral clusters using vCon- TACT2 (v.0.11.1)^112^ with the options ‘–rel-mode Diamond –db ProkaryoticViralRefSeq207-Merged–pcs-mode MCL –vcs-mode ClusterONE’. Viral family information was also assigned to contigs using Demovir (https://github.com/feargalr/Demovir). Where vContact2 was able to cluster viral contigs with a known virus, the taxa information of the known virus was assigned to the contig, otherwise the information from Demovir was used. The lifestyle of each viral contig (that is, virulent vs temperate) was predicted using BACPHLIP (v.0.9.6)^113^. If BACPHLIP assigned a lifestyle with a confidence value of ≥0.95, that lifestyle was assigned to the contig, otherwise, the lifestyle of the contig was denoted as ‘unassigned’. Putative bacterial host assignments for each contig were achieved with iPHoP (v.1.1.0)^72^ using the ‘Sept_2021_pub’ host database and default ‘predict’ parameters.

Quantitative analysis of the faecal virome inocula was achieved by first mapping the per-sample reads passing trimming and filtering onto the final database of putative viral contigs using Bowtie (v.2.4.5)^114^ in ‘–end-to-end’ mode. Read coverage and mapping counts were then collected using SAMTools (v.1.15.1)^115^. Spurious read mapping counts to viral contigs were eliminated from the count data by setting mapped read counts to 0 for any viral contig found to have 1x coverage over <75% of its length. Putative viral contigs were included in the quantitative analysis only if: the contig had been determined to have ≥50% completeness by CheckV, the number of CheckV-identified viral genes was ≥1 and the number of genes classified as ‘viral’ by CheckV was ≥10% of the total gene count. For all samples, the abundance of each viral contig in a sample was estimated by normalizing the number of reads mapping to a contig by the length of the contig, followed by normalization for library size via ‘total-sum scaling’^116^ (that is, length-normalized read count / total number of reads used for mapping in a sample). Absolute viral counts were calculated by comparing the relative abundances of normalized read counts to that of the spiked lactococcal phage Q33 contig. GBMs and GMMs were screened for within the assembled virome sequences by first annotating viral genes using eggNOG-mapper v.2.1.11, then searching the output KO against the GBM database.

### Analysis of total viral sequences within the bulk metagenomic sequencing data

Quantitative analysis of the total viral sequence information within the bulk metagenomic sequencing data followed a similar process used in the analysis of viral gavage samples. Namely, reads that passed trimming and filtering by fastp for each sample were mapped to the database of putative viral contigs obtained from the gavage samples as described above. Mapping results were then converted to total-sum scaled read counts as described above. Again, only those viral contigs that passed the CheckV filtering conditions described earlier were included in read count table.

### Bioinformatics and statistical analysis of microbiome data

Further data handling of microbiome measures was undertaken in R (v.4.2.2) using the Rstudio GUI (v.2022.7.2.576).

Interplate biases were recalibrated using the Metacal package in R^117^. Therefore, taxa with a minimal count of <5 were excluded from the bacterial analysis and taxa with a prevalence of <50% were excluded from the phage analysis, as ratios are invariant to subsetting and this study employs compositional data analysis techniques^118,119^. This approach was used since phages are heterogeneously distributed among microbiomes and if a feature is absent (or unobserved) in approximately half of samples, then inclusion in subsequent analysis greatly increases the risk of spurious associations^105^. Principal component analysis was performed on centred log-ratio transformed (clr) values. Zeroes were replaced using the ‘const’ approach described in ref. ^120^. Beta-diversity was computed in terms of Aitchison distance or Euclidean distance between clr-transformed data and assessed by permutational multivariate analysis of variance (PERMANOVA) using the vegan package. Alpha-diversity was computed using the iNEXT library^121^.

Differential microbial abundance was assessed using generalized linear mixed-effects models using the lme4 framework^122^. Custom functions and scripts are available online at https://github.com/thomazbastiaanssen/Tjazi. Where appropriate, post hoc pairwise comparisons were performed using one-tailed Tukey’s procedures with a *P* value cut-off of 0.05, in addition to a *P* value for the orthogonal restoration comparisons with a threshold of 0.05 and a Benjamini–Hochberg adjusted *q*-value cut-off of 0.2.

Restoration of microbial taxa was assessed using orthogonal contrasts in a generalized linear mixed-effects model, weighing the ‘Ctr’ and ‘FVT Stress’ group against the ‘Ctr Stress’ group. Figures were generated using ggplot2, ggforce and patchwork in R.

### Phage epifluorescence quantification

VLPs in the FVT solution were stained with SYBR gold as adapted from a previous method^123^. Briefly, the FVT solution was diluted and passed through an all-glass vacuum filter onto an Anodisc (25 mm diameter, 0.02 μm pore size; Whatman) for 60 s. Then, the Anodisc was stained with SYBR gold for 20 min, antifade was added and it was then allowed to dry in the dark. Images were taken on a fluorescence upright microscope (Olympus BX53 Upright Research Microscope) at ×100 magnification, and VLPs in images were quantified using a Python script (available at https://zenodo.org/records/10460215).

### Transcriptomic sequencing

Hippocampus and amygdala RNA was extracted using the mirVANA kit (Qiagen). Messenger RNA sequencing was conducted by Source Bioscience on the Illumina NovaSeq 6000 (S4 flow cell, 150 bp paired-end lane yielding 2.5 billion read pairs). A reference genome of obtained sequences was created using the reference annotation: *Mus musculus* (organism), GRCm38, UCSC, genome browser (GRCm38.p6), Ensemble^124^. Reads were assessed and filtered for quality with fastqc using default parameters. Genes were annotated and counted with kallisto^125^ using default parameters.

### Differential gene expression and GO term enrichment analyses

Further handling of transcriptomic data was undertaken in R (v.4.2.2) using the Rstudio GUI (v.2022.7.2.576). Principal component analysis was performed on clr-transformed values. Zeroes were replaced using the ‘const’ approach described in ref. ^120^. Differences in overall gene expression were computed in terms of Aitchison distance (Euclidean distance between clr-transformed data) and assessed by PERMANOVA using the vegan package.

Differential gene expression was assessed using generalized linear models employing the base R stats lm framework. Custom functions and scripts are available online at https://github.com/thomazbastiaanssen/Tjazi refs. ^126,127^. Before statistical testing, hippocampal gene transcripts were filtered by variance using the genefilter library in R^128^, with 0.5 set as the cut-off. Restoration of gene expression was assessed using orthogonal contrasts in a generalized linear model, weighing the ‘Ctr’ and ‘FVT Stress’ groups against the ‘Ctr Stress’ group for the hippocampus and amygdala individually.

To assess whether a subset of genes were altered by stress and restored to control levels post FVT, we employed the enrichR framework^129^ on the GO database^130^. In parallel, we identified seven terms in the gene ontology database that corresponded with the objectives of the study design. These seven terms were additionally assessed for enrichment using the base R stats phyper implementation of the hypergeometric test. Figures were generated using ggplot2 and patchwork in R.

### Statistical analyses

16S rRNA, metagenomic and transcriptomic data were analysed using R, and the remaining statistical analyses were carried out using SPSS v.28. Measurements were taken from distinct samples and not measured repeatedly. In outputs where multiple technical replicates were performed (for example, corticosterone and inflammatory cytokine analysis), the mean per individual was used. The restoration by FVT of features altered by stress was analysed by planned orthogonal contrast (*P*), followed by Benjamini–Hochberg’s adjusted false discovery rate (BH-FDR) of the calculated *P* value (*q*) and Tukey’s post hoc test (*P*; pairwise comparison of Ctr–Ctr Stress, followed by comparison of Ctr Stress–FVT Stress). For the phage–host network (Extended Data Fig. 5), the Anansi package^131^ was used whereby data were compared using linear models, followed by BH-FDR and Pearson’s correlation coefficient to plot the phage–host pairs. Unless otherwise noted, significant effects are denoted by: *P < 0.05, **P < 0.01, ***P < 0.001 and †*q* < 0.2. Technical outliers were removed before statistical analyses (for example, animals that did not complete tests, samples not available for assays, values outside limits of detection and so on). Values that were 2.5 standard deviations from the mean were considered statistical outliers and excluded from the related analyses (maximum of 2 statistical outliers per test). Data distribution was assumed to be normal, but this was not formally tested. Analyses were conducted in a manner blinded to the experimenter.

### Reporting summary

Further information on research design is available in the Nature Portfolio Reporting Summary linked to this article.

## Supplementary information

Source data

## Figures and Tables

**Extended Data Table 1:**
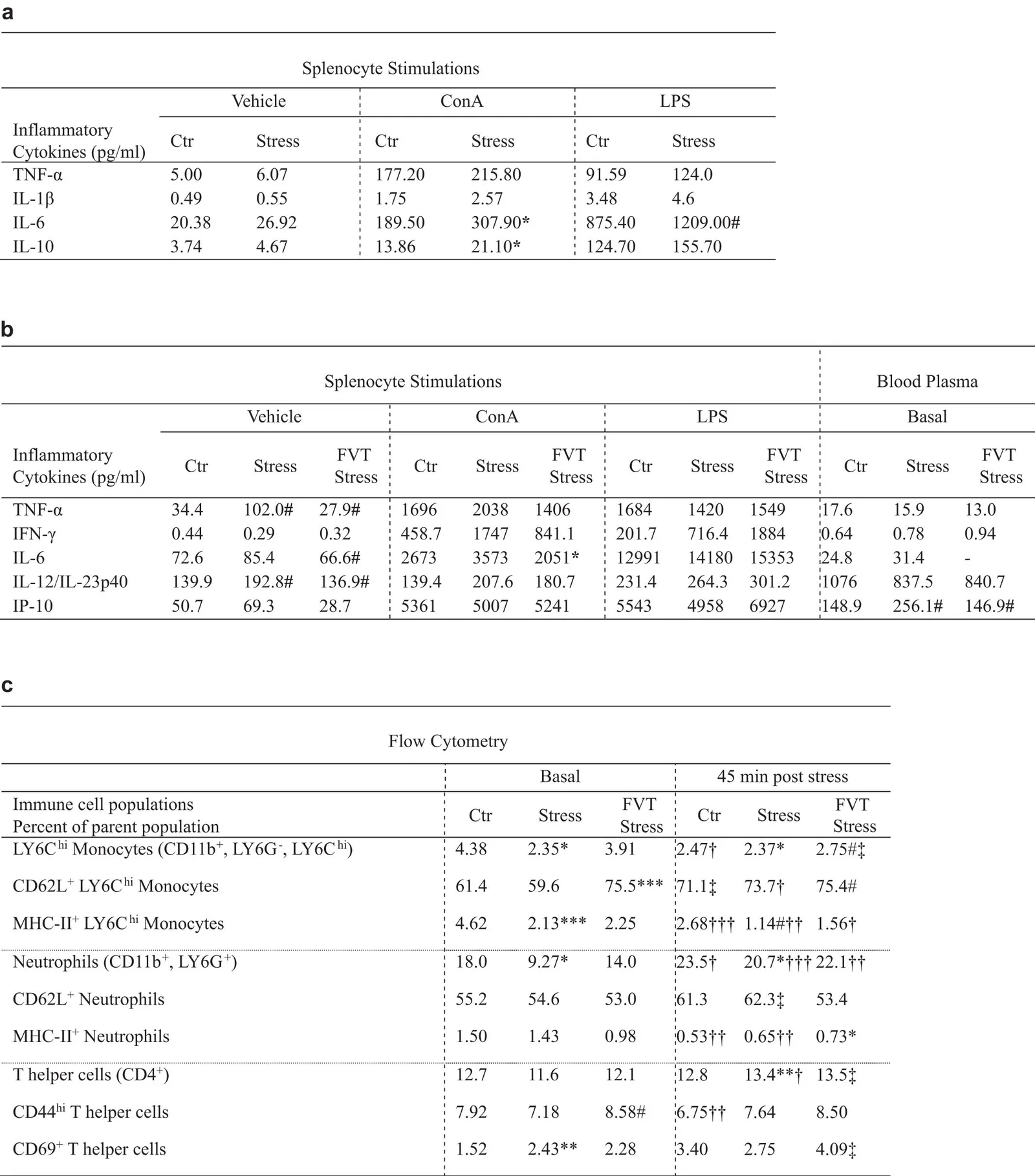
Inflammatory cytokine and circulating immune cell population data

data 45-min post stress is additionally tested by paired-sample T-test to basal data (†††*p* < 0.001, ††*p* < 0.01, †*p* < 0.05, ‡*p* < 0.1) (*n* = 6–10 per group for each measure).

**Extended Data Table 2:**
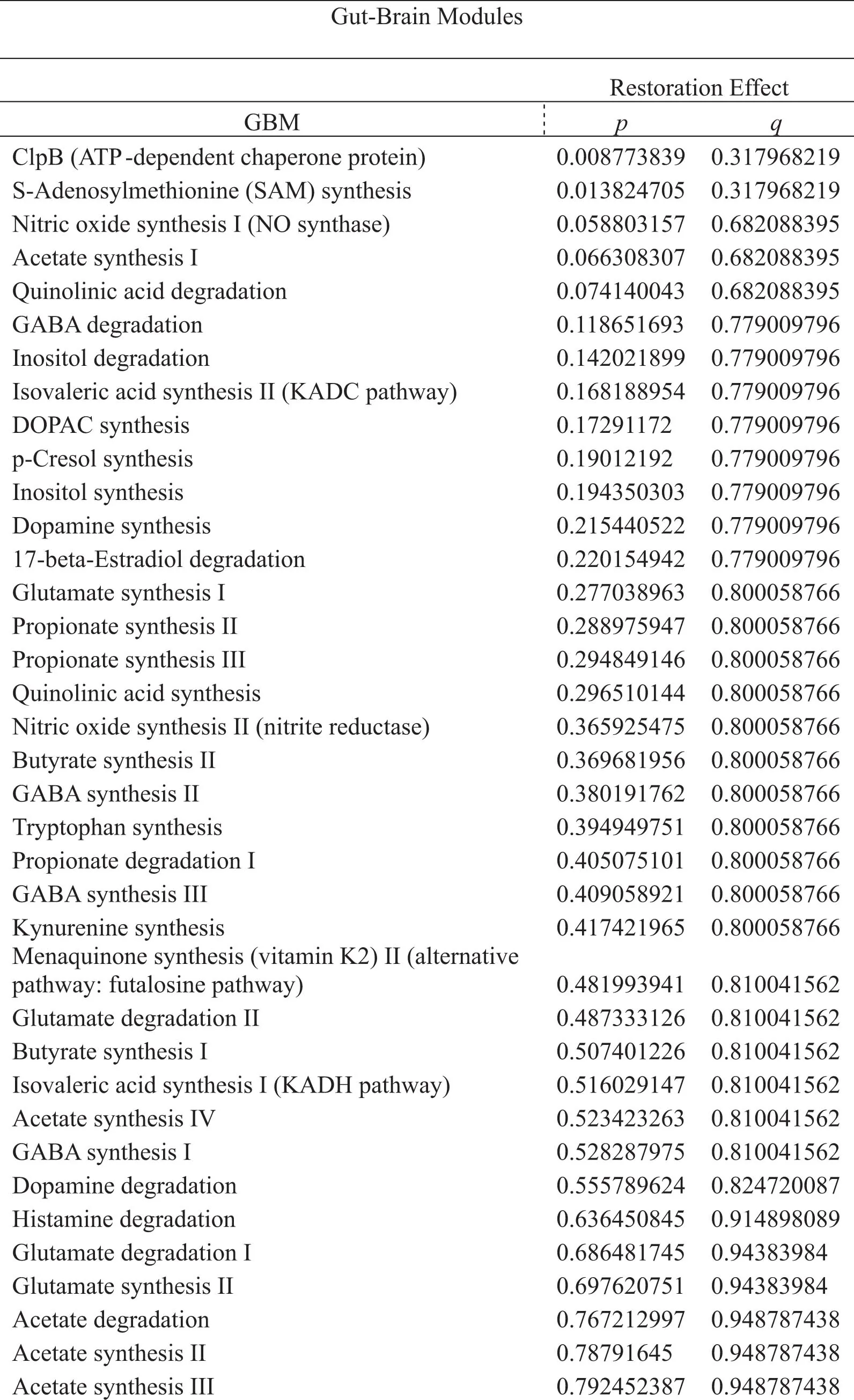
FVT gut-brain modules

The faecal bacteriome was assessed for carriage of gut-brain modules (GBMs) at the end of the study. The restoration effect was measured for each GBM altered by stress and restored by FVT by linear models (*p*-value) and Storey’s false discovery rate was performed for all measures (*q*-value).

**Extended Data Table 3:**
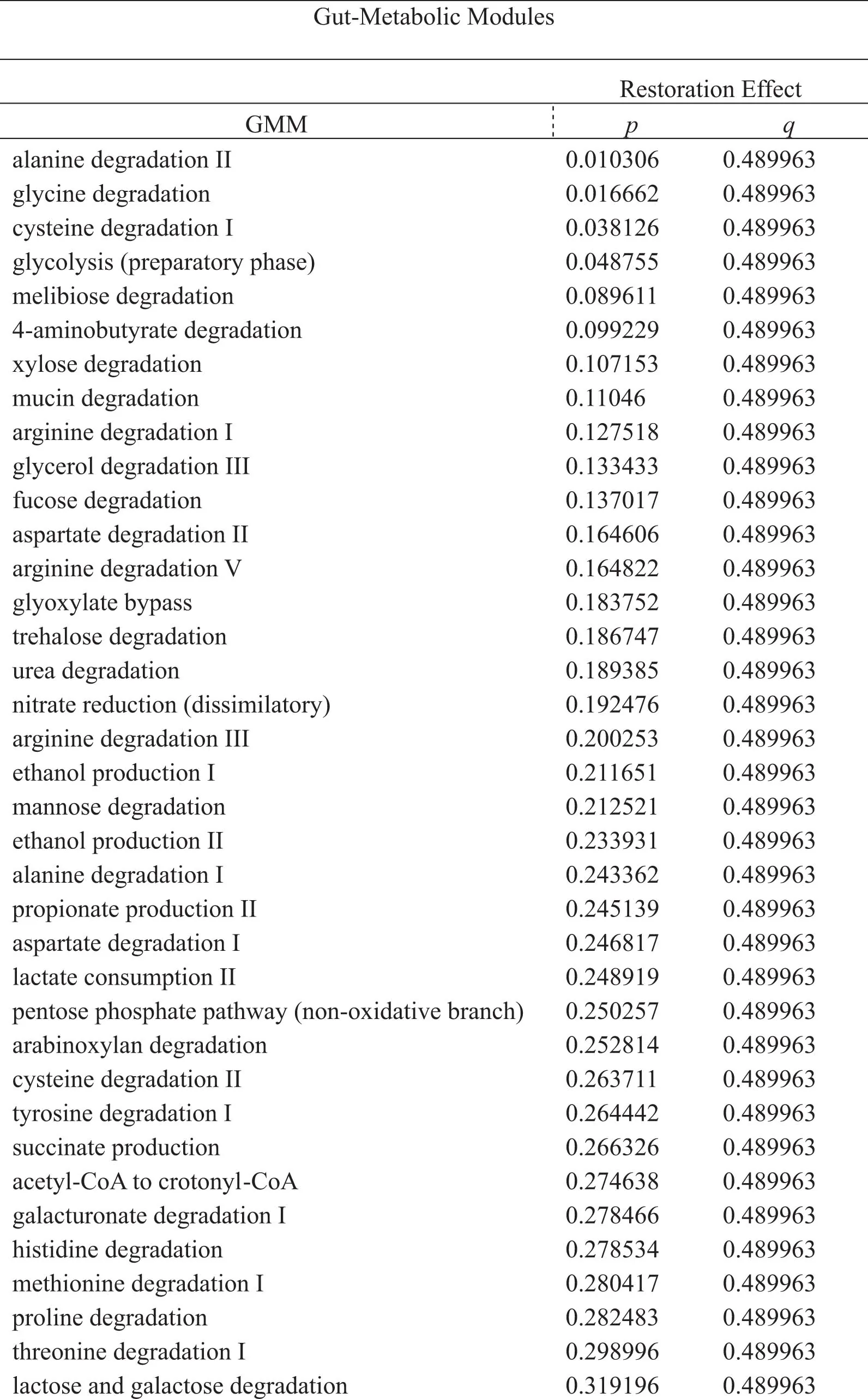
FVT gut-metabolic modules

The faecal bacteriome was assessed for carriage of gut- metabolic modules (GMMs) at the end of the study. The restoration effect was measured for each GMM altered by stress and restored by FVT by linear models (*p*-value) and Storey’s false discovery rate was then performed for all measures (*q*-value).

## Data Availability

16S rRNA sequencing data can be found in the European Nucleotide Archive (ENA) under accession PRJEB69687. Metagenomic sequencing data can be found in the Sequence Read Archive (SRA) of NCBI under BioProject accession PRJNA970614. Transcriptomic sequencing data can be found in ENA under accession PRJEB67706. Source data that support the findings of this study are available as supplementary data.

## Code availability

The scripts used for processing and generating the 16S rRNA, metagenomic and transcriptomic sequencing datasets can be found on Zenodo (https://doi.org/10.5281/zenodo.10144096). Additional material describing the microbiome conceptual bioinformatic approaches used in this study can be found in refs. ^126,127^.
