# Supplementary material for: The gut virome is associated with stress-induced changes in behaviour and immune responses in mice
Source: Nat Microbiol. 2024 Feb 5;9(2):359–76. doi: 10.1038/s41564-023-01564-y (PMC10847049; doi:10.1038/s41564-023-01564-y)
Supplement: Supplementary file 1 — Reporting Summary [file 41564_2023_1564_MOESM1_ESM.pdf]

nature portfolio

Corresponding author(s): John F Cryan

Last updated by author(s): Aug 5, 2023

Reporting Summary

Nature Portfolio wishes to improve the reproducibility of the work that we publish. This form provides structure for consistency and transparency in reporting. For further information on Nature Portfolio policies, see our Editorial Policies and the Editorial Policy Checklist.

Statistics

For all statistical analyses, confirm that the following items are present in the figure legend, table legend, main text, or Methods section.

n/a	Confirmed	
☐	☒	The exact sample size (n) for each experimental group/condition, given as a discrete number and unit of measurement
☐	☒	A statement on whether measurements were taken from distinct samples or whether the same sample was measured repeatedly
☐	☒	The statistical test(s) used AND whether they are one- or two-sided<br>*Only common tests should be described solely by name; describe more complex techniques in the Methods section.*
☐	☒	A description of all covariates tested
☐	☒	A description of any assumptions or corrections, such as tests of normality and adjustment for multiple comparisons
☐	☒	A full description of the statistical parameters including central tendency (e.g. means) or other basic estimates (e.g. regression coefficient) AND variation (e.g. standard deviation) or associated estimates of uncertainty (e.g. confidence intervals)
☐	☒	For null hypothesis testing, the test statistic (e.g. F, t, r) with confidence intervals, effect sizes, degrees of freedom and P value noted<br>*Give P values as exact values whenever suitable.*
☒	☐	For Bayesian analysis, information on the choice of priors and Markov chain Monte Carlo settings
☒	☐	For hierarchical and complex designs, identification of the appropriate level for tests and full reporting of outcomes
☒	☐	Estimates of effect sizes (e.g. Cohen's d, Pearson's r), indicating how they were calculated

Our web collection on statistics for biologists contains articles on many of the points above.

Software and code

Policy information about availability of computer code

Data collection	G*Power (version 3.1), Ethovision XT (version 11.5; Noldus), BD FACS Diva (version 8.0), BD CellQuest Pro (version 5.2, BD Biosciences), MESO QuickPlex SQ 120 SECTOR Imager 2400 (MSD), BX53 Upright Research Microscope (Olympus)
Data analysis	Flow cytometry data were analysed using FlowJo (v10).<br><br>Bacteriome sequencing data were analyzed using using FastQC (v0.11.9), Bowtie2 (2.3.5.1), Kneaddata (v0.10.0), Woltka (v0.1.3), Gomixer (omixer-rpmR v0.3.3), Metacal R package (https://github.com/elifesciences-publications/metacal), iNext (v3.0.0), eggNOG-mapper (v2.1.11).<br><br>Virome sequencing data were analyzed using fastp (v0.23.2), Kraken (v2.1.2), FastQC (v0.11.9), MEGAHIT (v1.2.9), BLASTn (v2.12.0+), NCBI RefSeq database release 211, the Gut Virome Database, the JGI IMG/VR (v3, release #5, 12-10-2020), inhouse crAss-like bacteriophage genome database using BLASTn (v2.12.0+), Prodigal (v2.6.3), PHROGs (release v3), HMMER (v3.3.2), VirSorter2 v2.2.3, metAgenomes tool (VRCA; https://github.com/alexcritschristoph/VRCA), barrnap v0.9 (https://github.com/tseemann/barrnap), CheckV (v1.0.1; sequence database v1.2), vCon- TACT2 (v0.11.1), Demovir (https://github.com/feargalr/Demovir), BACPHLIP (v0.9.6), iPHoP (v1.1.0), Bowtie (v2.4.5), SAMTools (v1.15.1),<br><br>Total packages used for sequencing data sets were R (v4.2.2) and Rstudio GUI (v2022.7.2.576) using the packages ade4 (v1.7.20), annotate (v1.74.0), AnnotationDbi (v1.58.0), ape (v5.6.2), assertthat (v0.2.1), backports (v1.4.1), beeswarm (v0.4.0), Biobase (v2.56.0), BiocGenerics (v0.42.0), biomformat (v1.24.0), Biostrings (v2.64.1), bit (v4.0.5), bit64 (v4.0.5), bitops (v1.0.7), blob (v1.2.3), boot (v1.3.28), broom (v1.0.2), cachem (v1.0.6), cellranger (v1.1.0), cli (v3.6.0), cluster (v2.1.4), codetools (v0.2.19), colorspace (v2.0.3), crayon (v1.5.2), data.table (v1.14.6), DBI (v1.1.3), dbplyr (v2.3.0), dplyr (v1.0.10), ellipsis (v0.3.2), fansi (v1.0.3), farver (v2.1.1), fastmap (v1.1.0), forcats (v0.5.2), foreach (v1.5.2), fs (v1.5.2), gargle (v1.2.1), genefilter (v1.78.0), generics (v0.1.3), GenomeInfoDb (v1.32.4), GenomeInfoDbData

(v1.2.8), ggbeeswarm (v0.7.1), ggforce (v0.4.1), ggplot2 (v3.4.0), ggrepel (v0.9.2), ggsankey (v0.0.99999), glue (v1.6.2), googledrive (v2.0.0), googlesheets4 (v1.0.1), gtable (v0.3.1), haven (v2.5.1), hms (v1.1.2), httr (v1.4.4), igraph (v1.3.5), iNEXT (v3.0.0), IRanges (v2.30.1), iterators (v1.0.14), jsonlite (v1.8.4), KEGGREST (v1.36.3), knitr (v1.41), lattice (v0.20.45), lifecycle (v1.0.3), lme4 (v1.1.29), lmerTest (v3.1.3), lubridate (v1.9.0), magrittr (v2.0.3), MASS (v7.3.58.2), Matrix (v1.5.3), memoise (v2.0.1), metacal (v0.2.0.9010), metafolio (v0.1.1), mgcv (v1.8.41), minqa (v1.2.5), modelr (v0.1.10), multtest (v2.52.0), munsell (v0.5.0), nlme (v3.1.162), nloptr (v2.0.3), numDeriv (v2016.8.1.1), patchwork (v1.1.2), permute (v0.9.7), phyloseq (v1.40.0), pillar (v1.8.1), pkgconfig (v2.0.3), plyr (v1.8.8), png (v0.1.8), polyclip (v1.10.4), purrr (v1.0.1), R6 (v2.5.1), Rcpp (v1.0.9), RCurl (v1.98.1.9), readr (v2.1.3), readxl (v1.4.1), reprex (v2.0.2), reshape2 (v1.4.4), rhdf5 (v2.40.0), rhdf5filters (v1.8.0), Rhdf5lib (v1.18.2), rlang (v1.0.6), RSQLite (v2.2.20), rstudioapi (v0.14), rvest (v1.0.3), S4Vectors (v0.34.0), scales (v1.2.1), sessioninfo (v1.2.2), stringi (v1.7.12), stringr (v1.5.0), survival (v3.4.0), tibble (v3.1.8), tidyr (v1.2.1), tidyselect (v1.2.0), tidyverse (v1.3.2), timechange (v0.2.0), Tjazi (v0.1.0.0), tweenr (v2.0.2), tzdb (v0.3.0), useful (v1.2.6), utf8 (v1.2.2), vctrs (v0.5.1), vegan (v2.6.4), vipor (v0.4.5), waldo (v0.4.0), withr (v2.5.0), xfun (v0.36), XML (v3.99.0.13), xml2 (v1.3.3), xtable (v1.8.4), XVector (v0.36.0), zlibbioc (v1.42.0).

For manuscripts utilizing custom algorithms or software that are central to the research but not yet described in published literature, software must be made available to editors and reviewers. We strongly encourage code deposition in a community repository (e.g. GitHub). See the Nature Portfolio guidelines for submitting code & software for further information.

Data

Policy information about availability of data

All manuscripts must include a data availability statement. This statement should provide the following information, where applicable:

- Accession codes, unique identifiers, or web links for publicly available datasets
- A description of any restrictions on data availability
- For clinical datasets or third party data, please ensure that the statement adheres to our policy

Metagenomic sequencing data can be found in Sequence Read Archive (SRA) NCBI under BioProject accession PRJNA970614. Transcriptomic sequencing data can be found in European Nucleotide Archive (ENA) under accession PRJEB67706. 16S rRNA sequencing data can be found in the supplementary material. Source data that support the findings of this study are available from the corresponding author upon request.

Human research participants

Policy information about studies involving human research participants and Sex and Gender in Research.

Reporting on sex and gender	N/A
Population characteristics	N/A
Recruitment	N/A
Ethics oversight	N/A

Note that full information on the approval of the study protocol must also be provided in the manuscript.

Field-specific reporting

Please select the one below that is the best fit for your research. If you are not sure, read the appropriate sections before making your selection.

☒ Life sciences ☐ Behavioural & social sciences ☐ Ecological, evolutionary & environmental sciences

For a reference copy of the document with all sections, see nature.com/documents/nr-reporting-summary-flat.pdf

Life sciences study design

All studies must disclose on these points even when the disclosure is negative.

Sample size	To ensure adequate sample size to detect changes, an a priori power analysis was performed using G*Power (version 3.1) considering a power of 0.95 (1-beta-error) and an alpha-error of 0.05.
Data exclusions	For corticosterone and blood cytokine analysis, samples which did not reached the detection limit were not included in the analysis. Measurements were taken from distinct samples and not measured repeatedly, in outputs where multiple technical replicates were performed (e.g. corticosterone and inflammatory cytokine analysis) the mean per individual was used. Technical outliers were removed prior to statistical analyses (e.g., animals that did not complete tests, samples not available for assays, etc.). Values that were 2.5 standard deviations from the mean were considered statistical outliers and excluded from the related analyses (maximum of 2 statistical outliers per test).
Replication	Experiments were repeated at least two times with independent biological replicates and all replications revealed similar results. These analyses include Blood Cytokine Multiplex ELISAs and plasma corticosterone ELISAs which were run in duplicates.
Randomization	Mice were randomly allocated to the experimental groups, ensuring an equal distribution of bodyweights among the groups. Additionally,

Randomization	samples were analyzed in random order.
Blinding	Analyses were conducted in a manner blinded to the experimenter.

Reporting for specific materials, systems and methods

We require information from authors about some types of materials, experimental systems and methods used in many studies. Here, indicate whether each material, system or method listed is relevant to your study. If you are not sure if a list item applies to your research, read the appropriate section before selecting a response.

Materials & experimental systems

n/a	Involved in the study
☐	☒ Antibodies
☒	☐ Eukaryotic cell lines
☒	☐ Palaeontology and archaeology
☐	☒ Animals and other organisms
☒	☐ Clinical data
☒	☐ Dual use research of concern

Methods

n/a	Involved in the study
☒	☐ ChIP-seq
☐	☒ Flow cytometry
☒	☐ MRI-based neuroimaging

Antibodies

Antibodies used	Flow Cytometry: rat anti-CD11b VFITC (2:71, Miltenyi, #130-113-243, clone: REA592), hamster anti-CD69 PE (2:71, Miltenyi, #130-115-575, clone: H1.2F3), rat anti-CD62L PE-Cy7 (2:71, BioLegend, #104418, clone: MEL-14), rat anti-Ly-6G PerCP-Vio700 (2:71, Miltenyi, 130-117-500, clone: REA526), recombinant anti-Ly-6C APC (2:71, Miltenyi, 130-111-917, clone: REA796), rat anti-MHC-II BV421 (2:71, BioLegend, #107632, clone: M5/114.15.2), rat anti-CD4 BV605 (2:71, BioLegend, #100548, clone: RM4-5), rat anti-CD44 BV786 (2:71, BD Biosciences, #563736, clone: IM7). Viable cells were identified by live/dead stain (Fixable Viability Stain 780 (APC-Cy7), 1:1000, BD Biosciences, #565388).
Validation	All antibodies were previously validated for the species and application (see citations provided on respective manufactures websites for the above outlined catalogue numbers; https://www.miltenyibiotec.com/, https://www.biolegend.com/, https://www.bdbiosciences.com/). Furthermore, appropriate positive and negative controls (as outlined by the manufacturer) including relevant tissue (e.g. spleen) from age-matched mice were used for the establishment of the panel and during the experiments.

Animals and other research organisms

Policy information about studies involving animals; ARRIVE guidelines recommended for reporting animal research, and Sex and Gender in Research

Laboratory animals	Male C57Bl/6J mice (n=50; Envigo, UK), 8-weeks of age on arrival, were used as experimental animals in this study. Male CD1 mice (n=70; Envigo, UK), 8-weeks of age on arrival, were used as non-experimental social aggressors.
Wild animals	No wild animals were involved in this study.
Reporting on sex	Only male mice were used in this study.
Field-collected samples	No field-collected samples were involved in this study.
Ethics oversight	All experiments were approved by the Animal Experimentation Ethics Committee of University College Cork (AE19130/P063) and conducted in accordance with the European Directive 86/609/EEC and the Recommendation 2007/526/65/EC.

Note that full information on the approval of the study protocol must also be provided in the manuscript.

Flow Cytometry

Plots

Confirm that:

☒ The axis labels state the marker and fluorochrome used (e.g. CD4-FITC).

☒ The axis scales are clearly visible. Include numbers along axes only for bottom left plot of group (a 'group' is an analysis of identical markers).

☒ All plots are contour plots with outliers or pseudocolor plots.

☒ A numerical value for number of cells or percentage (with statistics) is provided.

Methodology

Sample preparation	To assess immunity following chronic social stress and faecal virome transplant intervention, mice were subjected to behavioral assessments, and blood sample preparation was done as previously described with minor modifications (van de Wouw et al. 2020). Briefly, blood was collected by tail tipping using Eppendorf tubes containing 2.5µL 3% EDTA solution to prevent blood clotting. Blood was resuspended in each 10mL home-made red blood cell lysis buffer (15.5mM NH4Cl, 1.2mM NaHCO3, 0.01mM tetrasodium EDTA diluted in deionized water) for 3min. Blood samples were subsequently centrifuged (1500×g, 5min), plasma taken for cytokine analysis using the Proinflammatory Panel 1 (mouse) V-PLEX Kit (Meso Scale Discovery, Maryland, USA), and cells resuspended in PBS containing 1:1000 FVS780 (PE-Cy7) (BD Biosciences, 565388) and incubated for 15min at room temperature to distinguish live from dead cells. Samples were then centrifuged (1500×g, 5min) and each aliquot resuspended in 50µl BV staining buffer (BD Biosciences, 563794). All subsequent procedures were conducted on ice or at 4°C. For the staining procedure, 5µl of FcR blocking reagent (Miltenyi, 130-092-575) was added to each sample. Samples were subsequently incubated with a mix of antibodies for extracellular staining to investigate (a) innate immune cell populations focused on monocytes / neutrophils, and (b) adaptive immune cell populations focused on T-cells and incubated for 30min on ice. Samples for panel a (monocytes / neutrophils populations) were subsequently washed in staining buffer and fixed in 4% PFA for 30min on ice. Fixed samples were subsequently resuspended in the staining buffer and analyzed the subsequent day using the BD FACSCalibur. Data was analyzed using FlowJo (version 10). Target populations were normalized to the number of living cells.
Instrument	Sample preparations from blood were analyzed using BD FACSCelesta.
Software	Data was analyzed using FlowJo (version 10).
Cell population abundance	Purity was validated using the appropriate single stained and isotype controls.
Gating strategy	A panel of whole blood markers was used to assess circulating immune cell populations on the FACSCelesta. This approach enabled initial gating for singlets, followed by the selection of the peripheral blood mononuclear cells, and live cells (using FVS780). Neutrophils were selected based on LY6G+ (PerCP-Vio700-conjugated) and CD11b+ (FITC-conjugated) . Monocytes were selected as being Ly6G–, CD11b+ and LY6Chi (APC-conjugated), after which CD62L+ cells (Pe-Cy7-conjugated) were quantified.

☒ Tick this box to confirm that a figure exemplifying the gating strategy is provided in the Supplementary Information.
